# The Bro1-like domain-containing protein, AtBro1, modulates growth and abiotic stress responses in Arabidopsis

**DOI:** 10.3389/fpls.2023.1157435

**Published:** 2023-05-12

**Authors:** Syed Muhammad Muntazir Mehdi, Michal Wojciech Szczesniak, Agnieszka Ludwików

**Affiliations:** ^1^ Department of Biotechnology, Institute of Molecular Biology and Biotechnology, Faculty of Biology, Adam Mickiewicz University in Poznan, Poznan, Poland; ^2^ Institute of Human Biology and Evolution, Faculty of Biology, Adam Mickiewicz University in Poznan, Poznan, Poland

**Keywords:** Bro1 like-domain, ABA, abiotic stress, seed germination, *Arabidopsis thaliana*

## Abstract

Abscisic acid (ABA) affects plant physiology by altering gene expression, enabling plants to adapt to a wide range of environments. Plants have evolved protective mechanisms to allow seed germination in harsh conditions. Here, we explore a subset of these mechanisms involving the *AtBro1* gene, which encodes one of a small family of poorly characterised Bro1-like domain-containing proteins, in *Arabidopsis thaliana* plants subjected to multiple abiotic stresses. *AtBro1* transcripts were upregulated by salt, ABA and mannitol stress, while *AtBro1*-overexpression lines demonstrated robust tolerance to drought and salt stress. Furthermore, we found that ABA elicits stress-resistance responses in loss-of-function *bro1-1* mutant plants and AtBro1 regulates drought resistance in Arabidopsis. When the *AtBro1* promoter was fused to the β-glucuronidase (GUS) gene and introduced into plants, GUS was expressed mainly in rosette leaves and floral clusters, especially in anthers. Using a construct expressing an AtBro1-GFP fusion protein, AtBro1 was found to be localized in the plasma membrane in Arabidopsis protoplasts. A broad RNA-sequencing analysis revealed specific quantitative differences in the early transcriptional responses to ABA treatment between wild-type and loss-of-function *bro1-1* mutant plants, suggesting that ABA stimulates stress-resistance responses *via* AtBro1. Additionally, transcripts levels of *MOP9.5, MRD1, HEI10*, and *MIOX4* were altered in *bro1-1* plants exposed to different stress conditions. Collectively, our results show that AtBro1 plays a significant role in the regulation of the plant transcriptional response to ABA and the induction of resistance responses to abiotic stress.

## Introduction

Crop growth and productivity are markedly affected by multiple environmental factors such as climate change, salt accumulation, temperature extremes and drought. Over the past few decades, extensive research has been conducted on the molecular and physiological responses to abiotic stress in plants (Dubois and Inzé, 2020). Such research is crucial, particularly in light of the severe challenge represented by climate change to the development of sustainable agriculture at a time of significant population growth (Ramírez et al., 2009; Ahuja et al., 2010). Drought, which directly affects crop growth and productivity, is the one of the most antagonistic abiotic stresses (Le and McQueen-Mason, 2006; Zhu, 2016). High salinity also affects large areas of cultivated land and can be extremely damaging to crop plants: Na^+^, Ca^2+^ and Cl^–^ ions enter cells during salt stress, causing both ionic and osmotic stress in plants (Hu and Schmidhalter, 2005; Munns and Gilliham, 2015; Yang et al., 2015).

Phytohormones are induced by abiotic stress conditions such as drought. Molecular and cellular responses to stress involve plant hormones such as ABA, which activates signalling pathways, resulting in the expression of stress-related genes (Lee and Luan, 2012; Kuromori et al., 2018; Yoshida and Fernie, 2018) and the initiation of diverse physiological processes (Giraudat et al., 1994; Finkelstein et al., 2002; Yoshida et al., 2014; Harris, 2015; Sah et al., 2016; Vishwakarma et al., 2017). The core ABA signaling pathway is also vital for the regulation of plant growth and development In Arabidopsis, ABA receptors and three core components have been described. These include PYR/PYL/RCAR protein family members of the type 2C protein serine/threonine phosphatase (PP2C group A) and class III SNF-1-related protein kinase 2 (SnRK2) families (Mustilli et al., 2002; Yoshida et al., 2006; Fujii et al., 2007; Umezawa et al., 2009; Vlad et al., 2009; Lim et al., 2022). Plant growth under ideal conditions is promoted by SnRK2 kinases in the absence of ABA (Hasan et al., 2022). In Arabidopsis, the PP2Cs Abscisic Acid Insensitive 1 (ABI1) and Abscisic Acid Insensitive 2 (ABI2) are negative regulators of the ABA signaling pathway (Gosti et al., 1999; Merlot et al., 2001). Abiotic stress induces ABA biosynthesis-related genes such as *ABA1, ABA2, NCED3* and *AAO3* (Iuchi et al., 2001; Wan and Li, 2006), and in stomata the expression of *NCED3* is increased by ABA (Bauer et al., 2013; Leng et al., 2014).

Bro1 is a protein domain in which the N-terminal half adopts a boomerang or banana shape (Kim et al., 2005) and has been shown to have various functions. For example, in yeast (Odorizzi et al., 2003), the viability of *bro1* cells is reduced during nutrient starvation, resulting in defective regulation of cell proliferation (Nickas and Yaffe, 1996). Doa4, a ubiquitin thiolesterase that regulates membrane scission in endosomes, requires Bro1 for its recruitment to endosomal membranes (Luhtala and Odorizzi, 2004; Buysse et al., 2022). In anaerobic parasitic amoebozoans, multivascular body (MVB) formation during phagocytosis has been reported to be mediated by EhADH112, a Bro1 domain protein, on the cell surface and in endosomal compartments (Bañuelos et al., 2012; Pashkova et al., 2013). There are five Bro1-like domain-containing proteins in Arabidopsis, i.e. AT1G73390, BRAF, AT1G17940, ALIX and AT1G13310 (Kalinowska et al., 2015). The BRAF protein has been reported to regulate FYVE domain protein required for endosomal sorting 1 (FREE1) as well as MVB/prevacular compartment (PVC) function and degradation of membrane proteins (Shen et al., 2016).The apoptosis-linked gene-2 interacting protein X (ALIX/AtBro1) interacts with AMSH3 deubiquitinating enzyme and plays a role in its regulation (Kalinowska et al., 2015). Plants with mutations in *ALIX1* show a decrease in ABA hypersensitive responses and have compromised PYR/PYL/RCAR activity, leading to instability of the ABA receptor (García-León et al., 2019). ALIX protein is also implicated in vacuolar biogenesis where it participates in ESCRT-III complexes and affects vacuole morphogenesis (Cardona-López et al., 2015).

In our study, we investigated the molecular processes of the AT1G73390, which we have named as AtBro1 protein and we have shown response of AtBro1 in the presence of salt, mannitol and drought stress. ABA affected the levels of transcripts and localized in the plasma membrane. The loss-of-function mutant has shown less resistance to salt stress which affected seed germination and fresh weight than the WT Col-0 or overexpression lines. Therefore indicating that Arabidopsis AtBro1 prtein might be playing an essential role in regulating abiotic tolerance. Transcriptomic data analyses shows that AtBro1 regulates the expression of ABA-responsive genes negatively in the mutant of AtBro1. Overall, our study results demonstrate a novel function of AtBro1 in the transcriptional analysis and regulation of abiotic stresses.

## Materials and methods

### Plant materials and growth conditions

Surface-sterilized *A. thaliana* ecotype Columbia (Col-0) seeds were germinated on Murashige & Skoog medium (½ MS) containing 1% sucrose. After two-days stratification (4°C), the seeds on petri plates were transferred to a growth chamber and grown under conditions described previously (Ludwików et al., 2009). Ten-days-old seedlings were transferred to soil and grown at 22°C under long-day (LD) conditions (16 h light/8 h dark cycle). The *bro1-1* mutant (SALK_204462C), which has a T-DNA insertion between the sixth and seventh exons of *AtBro1* (At1G73390), was obtained from the Salk Institute Genomic Analysis Laboratory (SIGnAL) Collection at the Salk Institute (USA) (Alonso et al., 2003).

### Constructs and generation of transgenic plants

To generate an AtBro1-overexpressing construct, we used the *pENTR™/SD/D-TOPO* ^®^ vector. *Pfu* polymerase was used to amplify the entire open reading frame of AtBro1, which was cloned into the *pENTR* vector and was then sequenced as previously (Mitula et al., 2015b). The resultant clone was digested with *Mlu*I, and the cDNA-containing restriction fragments were recombined with the pEarleyGate 103 (Earley et al., 2006) vector using the Gateway^®^ LR Clonase^®^ II Enzyme Mix (Invitrogen), which resulted in the 35S:AtBro1-GFP construct, where the CaMV35S promoter provides constitutive expression in plant cells. The resulting AtBro1/pEarleyGate103 construct was verified by restriction mapping and DNA sequencing and the plasmid was transformed *via Agrobacterium tumefaciens* strain GV3101 into wild-type (WT) plants using the floral-dip method as described (Clough and Bent, 1998). The complementation lines 1 and 2 (Comp-1 and -2) were generated after crossing the *bro1-1* mutant with plants expressing 35S::AtBro1-GFP, resulting in constitutive expression of AtBro1. BASTA^®^ was used to select transgenic plants and the presence or absence of the transgene was inferred from their BASTA resistance or sensitivity. T3 transgenic plants were obtained for functional analysis. All primers used here are listed in **Supporting Information Table S1**.

### Subcellular localization of AtBro1 protein

The subcellular localization of AtBro1 protein in Arabidopsis protoplasts was determined as follows. Full-length coding sequences were cloned into pEarlyGate103, resulting in the 35S::AtBro1-eGFP construct, as detailed above. Leaf mesophyll protoplasts were isolated from three-week-old WT *A. thaliana* plants and transformed as described (Mitula et al., 2015a). A CBP20-RFP construct was simultaneously introduced and used as a transformation control, as well as a nuclear marker (Marczak et al., 2020). The lipophilic dye FM4-64, which incorporates into the outer leaflet of the plasma membrane where it emits an intense fluorescence between 580 nm and 650 nm, was also used: protoplasts were stained with a final concentration of 0.5% (*v/v*) of FM4-64 stock solution (1 μg/μl). After an incubation period of 10-15 min at room temperature, FM4-64 had spread to all areas of the plasma membrane in protoplasts (Blachutzik et al., 2012). Protoplasts transformed with the GFP and mRFP constructs were analyzed using a Nikon A1R confocal microscope as described (Marczak et al., 2020).

### Analyses of the AtBro1 T-DNA insertion line and overexpression lines

**Figure 1:**
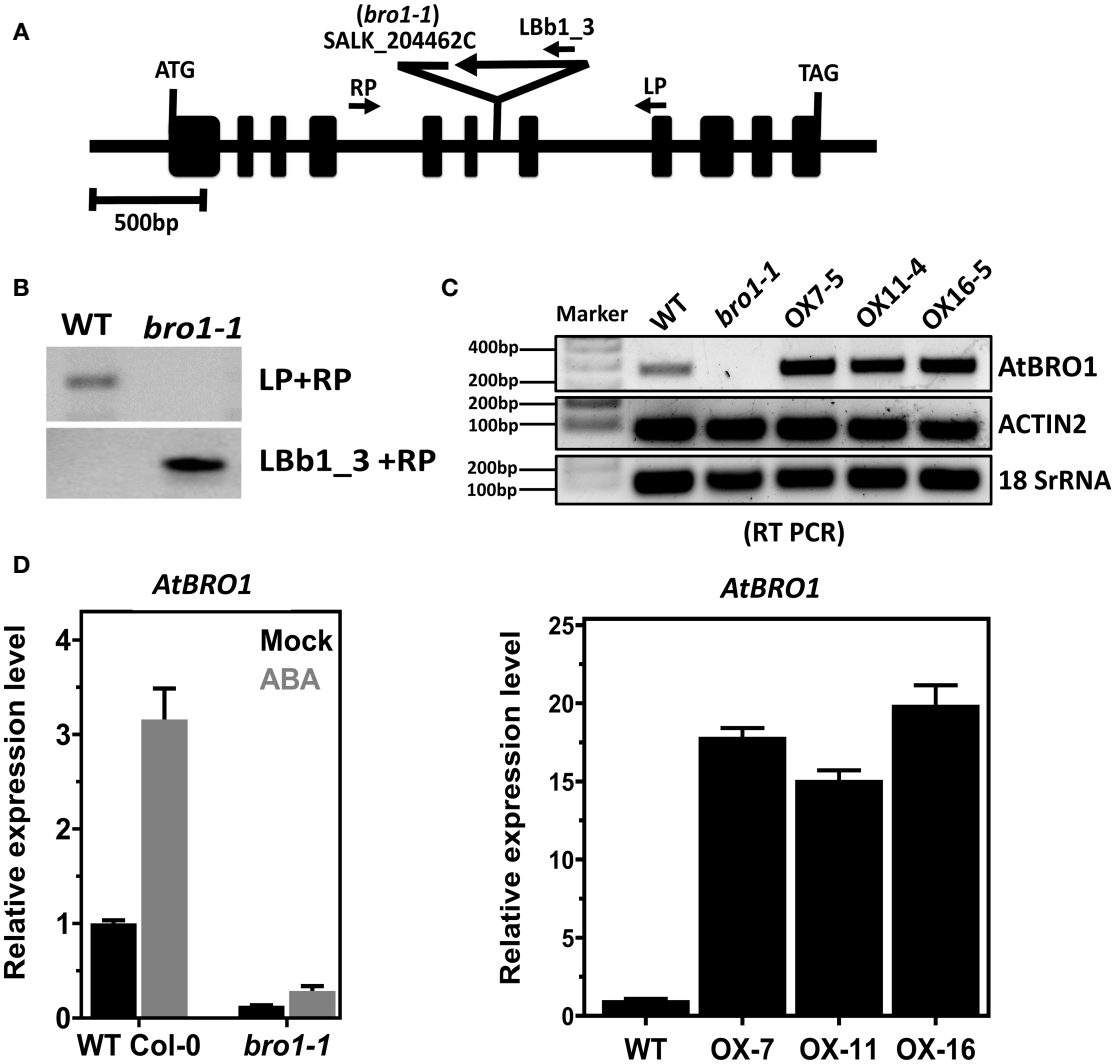
Identification of *bro1-1* mutant and overexpression transgenic lines. **(A)** Schematic diagram of the Arabidopsis AtBro1 gene with T-DNA insertion site. The exons are indicated by black boxes and the introns by black lines; the genotyping PCR primers are shown as arrows, in addition to denoting the T-DNA location. **(B)** Genomic PCR analyses using LBb1.3 and RP primers verified homozygosity of the T-DNA alleles along with insertion in the *bro1-1* mutant. **(C)** Semi-quantitative RT-PCR analysis of *AtBro1* transcripts in WT Col-0, *bro1-1* mutant and three independent transgenic OX lines of *AtBro1* (OX7-5, OX11-4 and OX16-5). *Actin2* and *18SrRNA* were used as internal controls. RT reactions were performed using primers specific for the *AtBro1* gene and amplified fragments were run on 1% agarose gels. **(D)** Quantitative RT-PCR (qRT-PCR) of *AtBro1* transcripts in two-week-old seedlings of WT Col-0 and the *bro1-1* mutant treated with or without (Mock) 100 μM ABA for 4 h, together with three independent transgenic *AtBro1*-OX lines (OX7-5, OX11-4 and OX16-5); the *actin2* gene served as an internal control.

The *bro1-1* mutant line with a T-DNA insertion was isolated from SALK insertion lines (SALK_204462C). PCR amplification with primers LP, RP and LBp1.3 (**Figure 1B**) was used to check the T-DNA insertion and subsequently to confirm the homozygous line. Primers were designed according to the SIGnAL website (http://signal.salk.edu/tdnaprimers.2.html). The semi-quantitative and qPCR analyses of the T-DNA insertion line and overexpression transgenic lines (OX) with or without 100 μM ABA (**Supporting Information Figure S1** and **Figures 1C, D** right) were performed as reported (Ludwików et al., 2009).

### Analysis of stress treatments and seed germination assays

Various abiotic stresses were applied to Arabidopsis plants as described (Tarte et al., 2015; Xing et al., 2020) with a few modifications. For stress treatments of WT prior to quantitative RT-PCR, 10-days-old seedlings grown on MS plates were treated with 300 mM NaCl and samples were taken at 0, 1, 2, 4 and 8 h. For the mannitol treatment, the 10-days-old WT seedlings were treated with 300 mM mannitol and sampled at 0, 2, 4, 8, and 12 h. For the ABA treatment, the 10-days-old WT seedlings were treated with 100 μM ABA for 0, 1, 2, 4 and 8 h. For the ABA treatment of WT, *bro1-1* mutant and OX lines prior to quantitative RT-PCR analysis, 50 μM ABA was used for 0 and 4 h on two-week-old seedlings grown on MS plates.

For the analysis of the salt-stress response, WT and *bro1-1* mutant seedlings were grown for four days on MS plates and then were transferred to MS plates with 0, 120, 130 and 140 mM NaCl added. For WT and AtBro1-OX transgenic plants, four-days-old seedlings grown on MS plates were transferred to MS plates with 0, 140, 150 and 160 mM NaCl. The fresh weight of seedlings was measured after seven days of salt treatment.

Seeds were horizontally germinated for the seed germination assay on MS medium with or without 150 mM NaCl, 250 and 300 mM mannitol, and 0.5 and 1 µM ABA for 7 or 8 days, when germination (emergence of radicles) and post-germination growth (green cotyledon appearance) were assessed.

### Drought stress and electrolyte leakage

Seedlings of WT, *bro1-1* and OX lines were grown on soil for three weeks with sufficient watering for the drought tolerance test. The plants were then exposed to drought by withholding irrigation; well-watered plants were used as controls. The same conditions were used for all pot trays, which were placed in a growth chamber; the position of each tray was randomly changed routinely to exclude any position effect during the drought stress experiment. The leaves of all plants tested began to wilt after withholding irrigation and when almost all plants of at least one genotype shown wilting such as *bro1-1* mutant line plants had shown wilting then we started to irrigate the plants again and allowed to grow for three more days. Photographs were taken after withholding water or re-watering and survival was calculated. Three independent experiments were carried out.

After drought stress, the detached leaves were incubated in deionized water for two hours and the conductivities (C1) of the solutions were measured as described (Liu et al., 2017) with a few modifications. The conductivity was determined using a conductivity meter (SevenMulti, Mettler Toledo, Warsaw, Poland). The leaves were then boiled for 15 min in the same deionized water. Then the solutions were allowed to cool and the conductivity (C2) of the resulting solutions was determined. The electrolyte leakage was calculated using the following formula: EL (%) = (C1/C2) X 100%.

### Construction of the *AtBro1* promoter–GUS vector for analysis of promoter activity

**Figure 2:**
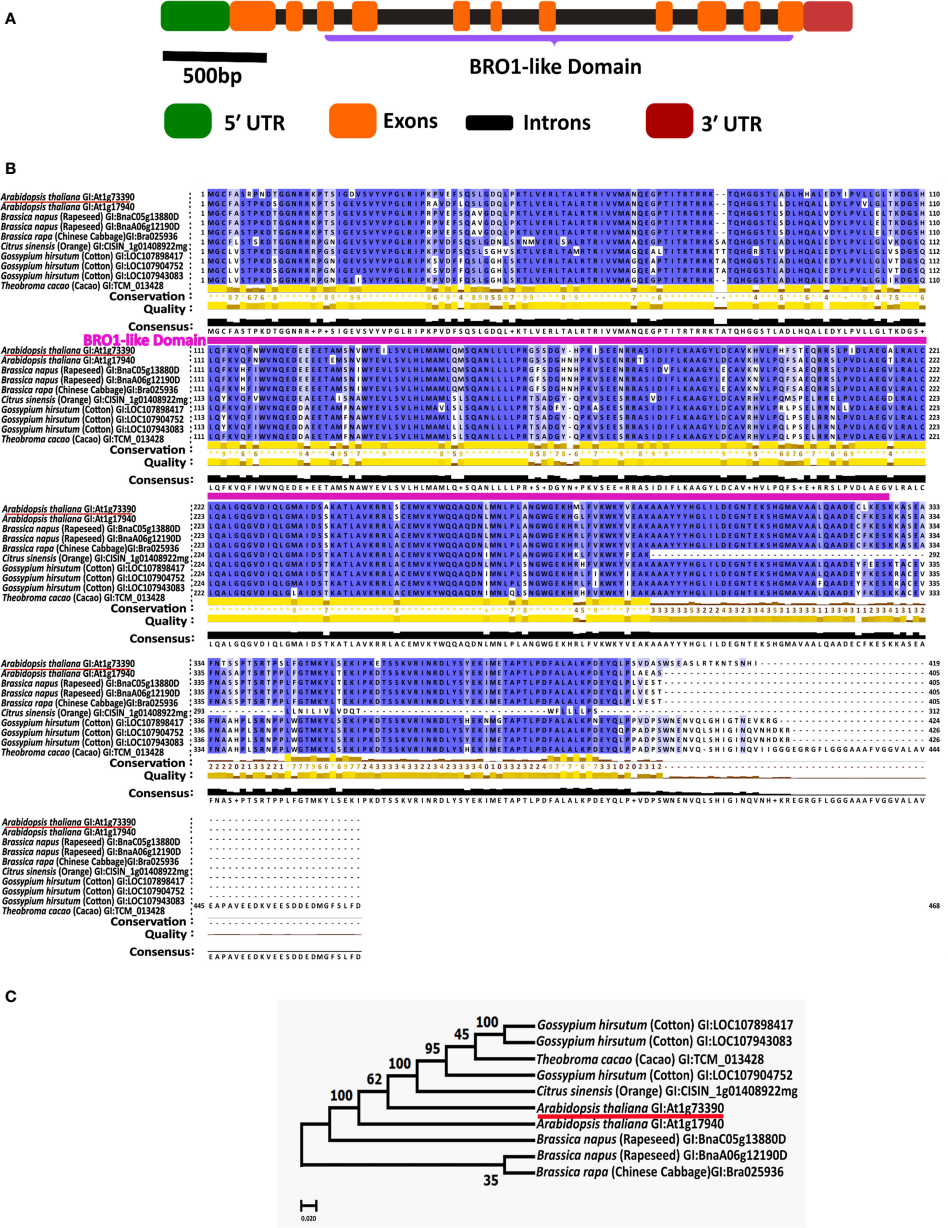
Protein domains of AT1G73390 gene and conservation of Bro1-like domain in a paralog and orthologs. **(A)** The exon-intron structure of the AT1G73390 gene showing the extent of its Bro1-like domain along with the untranslated regions. **(B)** Multiple sequence alignment was performed using CLUSTALW and conserved residues were identified using JalView 2.11.2.3 software. The conserved Bro1-like domain region is annotated. **(C)** Phylogenetic tree generated with the full-length ORFs in MEGA 10.2.2 software. The number at each node indicates the bootstrapped value for 1000 replicates. In B and C, the GI (Gene ID) numbers for each protein are shown.

To identify *cis*-regulatory elements, the nucleotide sequence of 1.5 kbp upstream from the start codon (ATG) of the *AtBro1* gene was obtained from the Arabidopsis Information Resource website (https://www.arabidopsis.org/index.jsp) and queried against PlantCARE (http://bioinformatics.psb.ugent.be/webtools/plantcare/html/) (Lescot et al., 2002) (**Figure 2C**).

To generate the *AtBro1* promoter-GUS fusion construct, the 1512-bp promoter region of the *AtBro1* gene was PCR amplified using the prom_AtBro1_GUS-F and prom_AtBro1_GUS-R primers (**Supporting Information Table S1**) and then cloned into the pMDC163 vector using the restriction enzymes *NcoI* and *XbaI* (Yang et al., 2015). The promoter sequence was verified by sequencing. The *A. tumefaciens* strain GV3101 carrying the pMDC163-proAtBro1::GUS construct was used to transform WT Col-0 plants using the floral-dip method (Clough and Bent, 1998). T3 homozygous lines of promAtBro1::GUS were used to assess promoter activity. Three representative lines showing the same expression pattern under all tested conditions were used for further detailed characterisation.

### Histochemical localization of GUS expression

T3 homozygous lines were grown on MS media for the aforementioned number of days. For histochemical analysis of GUS expression, two-week-old Arabidopsis seedlings were treated with or without 50 μM ABA for two hours. Tissue samples were submerged in a staining buffer [0.1% Triton X-100, 100 mM sodium phosphate, pH 7.0, 0.5 mM K_4_Fe(CN)_6_, 10 mM EDTA, 0.5 mM K_3_Fe(CN)_6_, 1 mM X-gluc], and incubated overnight at 37°C as mentioned previously (Mitula et al., 2015b). After the GUS reaction, plant samples were kept in 80% ethanol. A Zeiss Stereo Lumar V12 microscope was used to observe the GUS staining patterns in seedlings.

### RNA sequencing and data processing

Total RNA samples were extracted from two-week-old WT and *bro1-1* mutant seedlings with or without 100 μM ABA treatment for 2 and 4 h using an RNAeasy kit (Qiagen) according to the manufacturer’s instructions. A Bioanalyzer 2100 (Agilent, California, USA) was used to evaluate the total RNA integrity (RIN ≥ 7.5) and an RNA-sequencing library was constructed from this RNA and sequenced by the Macrogen Europe Company (Netherlands). The quality of the resulting FASTQ files was assessed with FastQC. Quality filtering and adapter clipping were carried out using BBDUK2 v37.02 using the following parameters: qtrim=w, maq=10, trimq=20, k=23, hdist=1, mink=11, minlength=100, removeifeitherbad=t, tpe, tbo. Additionally, reads that mapped to *A. thaliana* ribosomal RNAs were discarded with Bowtie 2 v2.3.5.1. Next, gene and transcript expression values were estimated with RSEM v1.2.30. Differential expression analysis was done with DESeq2 v1.34.0, using default parameters and a threshold of 0.05 for adjusted *P*-value. Differentially expressed genes (DEGs) were required to have a fold change in expression greater than 2 (upregulated genes) or less than 0.5 (downregulated genes). GO term enrichment of differentially expressed genes was done using ShinyGO 0.76.3 (http://bioinformatics.sdstate.edu/go/) (Ge et al., 2020), requiring that the calculated FDR was < 0.05.

### RNA extraction and RT–qPCR analysis

For the mRNA, sequence data validation and time course experiments were done as described (Ludwików et al., 2009). Total RNA was isolated using an RNAeasy kit following the manufacturer’s instructions (Qiagen). cDNA was synthesized using the Maxima first-strand cDNA synthesis kit with DNAse (Thermo Fisher Scientific) according to the manufacturer’s instructions. Total RNA (2-3 μg) was treated with DNase (included in Maxima first-strand cDNA synthesis kit, Thermo Fisher Scientific) and then first strand cDNA was synthesised using half of the DNase-treated. The other half of the DNase-treated RNA sample was used as a negative control. The manufacturer’s instructions for the SYBR Green Real-Time PCR Master Mix reagent (Thermo Fisher Scientific) were followed, and RT-PCR was performed on an Applied Biosystems QuantStudio™ 7 Flex Real-Time PCR System (Applied Biosystems) in a volume of 20 μl. Three biological replicates were performed in triplicate for each cDNA sample, negative control and no-template control. *18SrDNA* or *ACT2* was used as a reference. Data analyses were performed using QuantStudio Software (Applied Biosystems). After obtaining data values from the qPCR machine, the 2^ΔΔCt^ method was used to calculate relative expression levels (Livak and Schmittgen, 2001) and all gene expression levels were normalized against *18SrRNA* or *ACT2*. Results were averaged from three consistent experiments. Primers used in all qPCR assays are listed in **Supporting Information Table S1**.

### Multiple alignment analysis and phylogenetic tree

The amino acid sequences were aligned using Clustal Omega software (https://www.ebi.ac.uk/Tools/msa/clustalo/(access on 10^th^ June 2022) and then manually corrected. The maximum likelihood phylogenetic tree was constructed using MEGA 10.2.2 software; the numbers at each node indicate the bootstrapped values for 1000 replications.

### Statistical analyses

A two-tailed Student *t*-test was used to obtain *P*-values using Excel software to calculate the significance between samples. A value of *P*<0.05 was considered statistically significant. Asterisks indicate significant differences between the samples compared, as shown in the figure legends. Treatments and assays in this study were performed in at least three independent experiments. The values presented are the means ± SD in all experiments.

### Data availability

The data generated in this study are available as part of the article and its supplement. The RNA-Seq data have been deposited for public use in the Gene Expression Omnibus (GEO) under accession number GSE225062.

## Results

### The *AtBro1* gene encodes a bro1-like domain-containing protein

Our previous study identified several novel miRNAs whose expression was suppressed by ABA in WT plants and other genotypes (Mehdi et al., 2021). One of these miRNAs is thought to target AT1G73390 (*AtBro1*), a gene of unknown function whose role in the response to various abiotic stress factors we aimed to determine in the current work. *AtBro1* encodes a protein of 419 amino acids, including a single Bro1-like domain, and is one of a small family of five such proteins in the Arabidopsis genome (Kalinowska et al., 2015) (**Figure 2A**). Using BLASTP, we identified one paralog (encoded by *AT1G17940*) and numerous orthologs in *Brassica napus* (rapeseed), *Citrus sinensis* (orange), *Gossypium hirsutum* (cotton), and *Theobroma cacao* (cacao). The ClustalW2 program was used to perform multiple alignments of these amino acid sequences. A high degree of amino acid sequence conservation was observed, particularly in the Bro1-like domain (**Figure 2B**). To explain the phylogenetic relationships between AtBro1 and its paralog and orthologs, a phylogenetic tree was constructed. The paralog AT1G17940 was most similar to AtBro1, with the proteins encoded by *B. napus* genes being the most closely related among the orthologs (**Figure 2C**).

### *AtBro1* expression pattern in response to multiple stress factors

**Figure 3:**
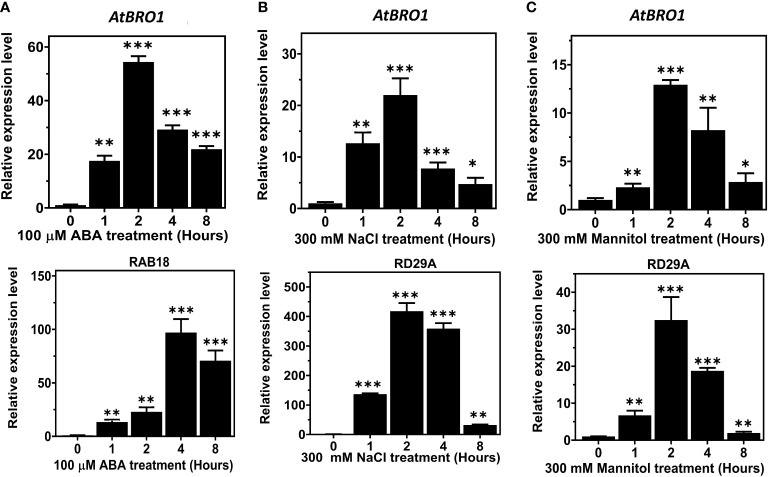
Expression analysis of AT1G73390 (AtBro1) in WT seedlings under abiotic stress conditions. **(A–C)** Quantitative RT-PCR (qRT-PCR) analysis of *AtBro1*, *RD29A*, and *RAB18* after treatment with 100 μM ABA, 300 mM NaCl and 300 mM mannitol for 0, 1, 2, 4 and 8 h. *RAB18* was used as a control for ABA treatment and *RD29A* as a control for NaCl and mannitol treatment. *18SrRNA* and *Actin2* genes were used as internal controls. The levels of transcripts at 0 h were set to 1. Values show standard deviation (n=3 biological replicates with independent technical triplicates for each). Significant differences were determined by Student’s *t*-test: ∗∗∗*P* < 0.001, ∗∗*P* < 0.01, ∗*P* < 0.05.

qRT-PCR revealed that *AtBro1* transcript levels in WT seedlings were significantly increased by exogenous ABA treatment. The response to ABA was rapid and a significant upregulation of *AtBro1* was observed after 1 h of treatment (**Figure 3A**). In addition, it was found that AtBro1 was also significantly upregulated by salt and mannitol stress (**Figures 3B, C**). Two genes, *RAB18* and *RD29A*, were used as positive controls for the different abiotic stresses. These results suggest that AtBro1 is involved in the response to the phytohormone ABA and to other abiotic stresses.

### GUS histochemical analysis and subcellular localization of AtBro1 protein

**Figure 4:**
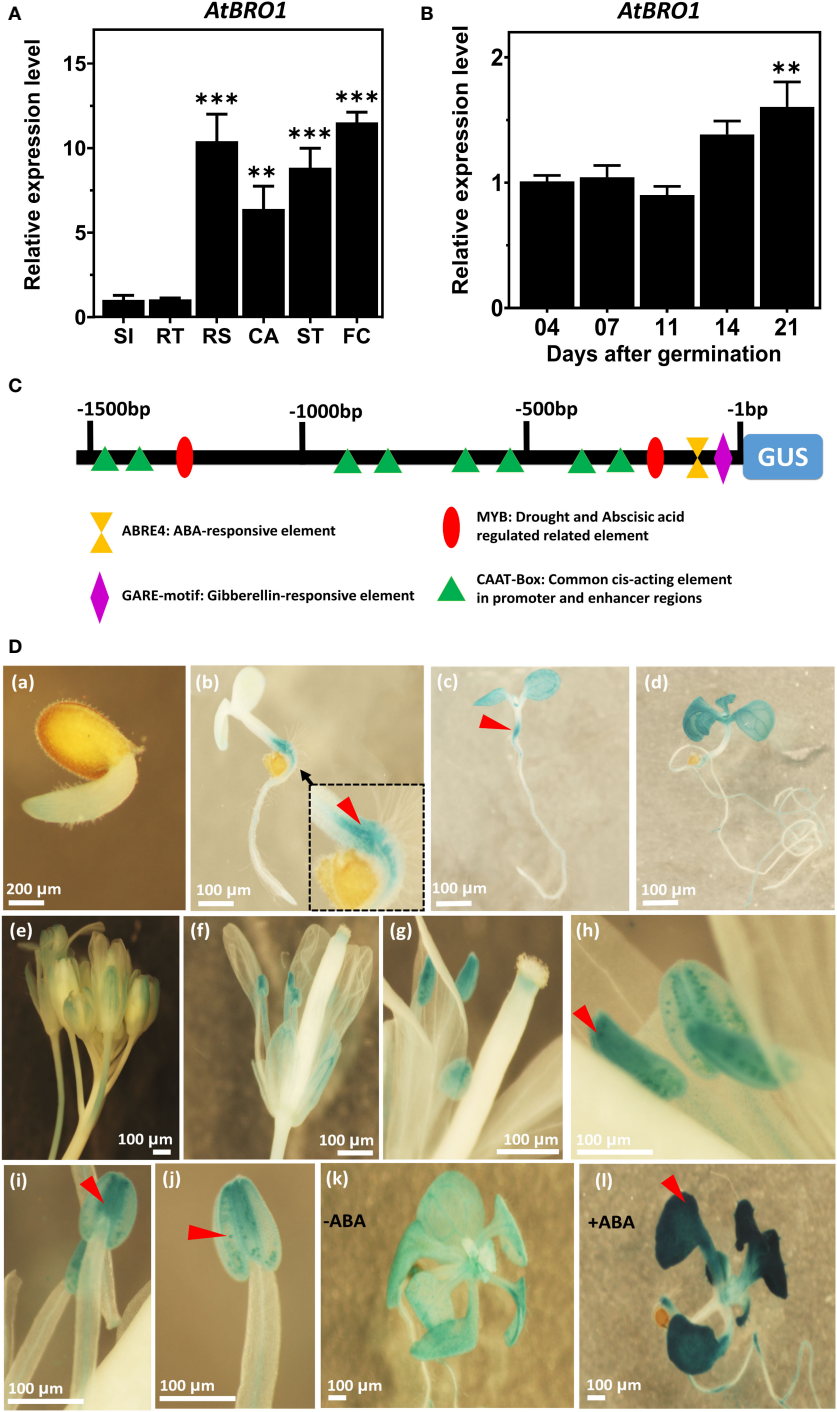
Temporal and spatial expression patterns of AtBro1. **(A)** Quantitative RT-PCR analysis of AtBro1 expression in 4-, 7-, 11-, 14-, and 21-days-old WT seedlings grown under SD conditions. *18SrRNA* and *Actin2* were used as internal controls. The transcript level four days after germination was set as 1. **(B)** Quantitative RT-PCR analysis of AtBro expression in organs of 43-days-old WT Col-0 plants. *18SrRNA* was used as internal control. The transcript level in siliques was set as 1. RS, rosette leaves; RT, roots; ST, stems; SI, siliques; FC, floral clusters; CA, cauline leaves. **(C)** Schematic map of the AtBro1 promoter used for tissue-specific expression of GUS in GUS vector pMDC163. *Cis*-elements present in the promoter region are shown in different shapes and colors. **(D)** GUS staining of different tissues of pAtBro1::GUS transgenic plants. Expression of pAtBro1::GUS in cotyledons and hypocotyl of 1, 4, 7 and 11-days-old seedlings **(a–d)**, inflorescence **(e)**, an open flower **(f)** and stamens **(g–j)** of mature plants grown under LD conditions. The pAtBro1::GUS seedlings were grown on ½MS medium. Two-week-old plants were treated either without ABA **(k)** or with 50 μM ABA for two hours **(l)**. Representative GUS staining results are shown. Scale bars are represented in all figures **(a–l)**. Significant differences were determined by Student’s t-test: ***P < 0.001, **P < 0.01.

To analyse the spatial and temporal expression patterns of AtBro1 during seedling development in WT Arabidopsis plants, we extracted total RNA from seedlings 4, 7, 11, 14 and 21 days after germination and performed qRT-PCR (**Figure 4A**). *AtBro1* transcript levels were generally high, and were highest after 21 days of seedling development. AtBro1 transcripts were detected at various growth stages and in different tissues, such as stems, leaves, roots, cotyledons, apical buds, flower buds and flowers, with significantly higher expression in leaves and flowers than in roots and siliques in 43-days-old WT Col-0 plants. The transcript levels in flower clusters and rosette leaves were more abundant than in other tissues of Arabidopsis plants (**Figure 4B**).

Next, we analysed the promoter sequence of *AtBro1* to identify the core and *cis*-regulatory elements. Both strands (plus and minus) of the *AtBro1* promoter sequence contained stretches with TATA-box elements (not shown in figure) and, at discrete locations, various CAAT boxes, along with other *cis*-regulatory elements such as ABRE4, GARE and Myb motifs. To determine the spatial and temporal patterns of *AtBro1* expression, we fused the 1512-bp promoter region to the β-glucuronidase (GUS) coding region and used this to generate pAtBro1::GUS transgenic plants (**Figure 4C**). GUS expression was then analysed in the true leaves, stems and cotyledons of 7-days-old seedlings, 11-days-old rosette leaves and roots. The expression pattern was consistent throughout all stages of plant development (**Figures 4D_a–d**). GUS expression was observed to be highest in floral tissues, especially in anthers, but was also high in cotyledon leaves. In true leaves, the low level of GUS staining in seedlings and at the flowering stage (**Figures 4D_e–j**) reflected the results obtained by qRT-PCR. Since AtBro1 transcription is markedly induced by ABA, we treated two-week-old seedlings with the phytohormone and observed higher GUS expression in the leaves of the seedlings (**Figures 4D_k, l**). These patterns of GUS expression were also consistent with the results of the qRT-PCR analysis. Together, these data suggest that AtBro1 is involved in both the vegetative and reproductive development of Arabidopsis.

**Figure 5:**
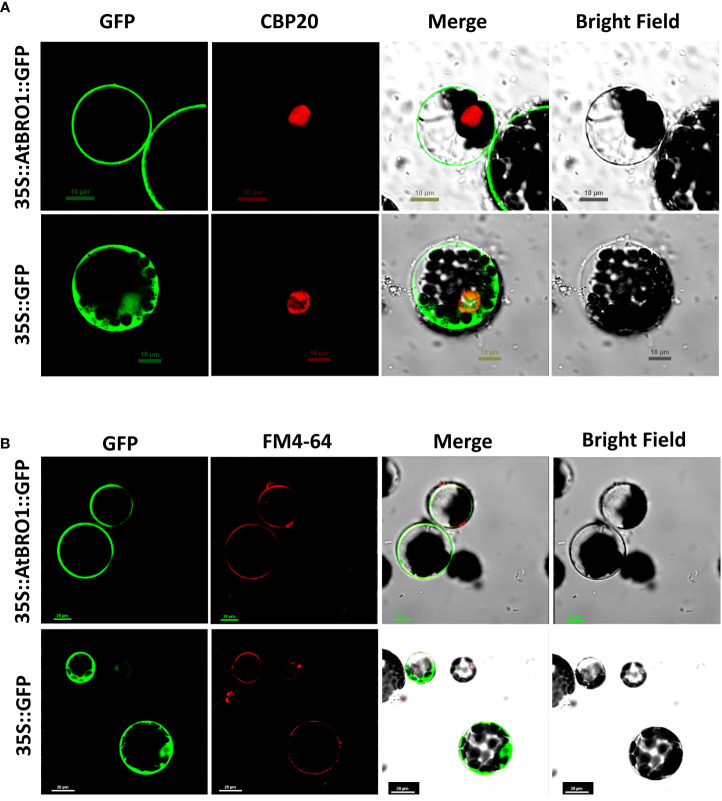
Subcellular localization of an AtBro1-GFP fusion protein. Expression of 35S::AtBro1-GFP in Arabidopsis protoplasts analyzed by confocal microscopy. **(A)** CBP20 fused with RFP was used as a nuclear marker. **(B)** Lipophilic dye FM4-64 was used as a plasma membrane marker. Empty vector was used as a negativecontrol. Scale bars = 10 *μ*m and 20 *μ*m.

To investigate the subcellular localisation of AtBro1, a C-terminal fusion of AtBro1 with green fluorescent protein (GFP) was generated and expressed in Arabidopsis protoplasts. AtBro1-GFP was found exclusively in the membrane of Arabidopsis cells (**Figure 5**). We then used a plasma membrane-specific control dye, FM4-64, to confirm the localization of AtBro1 to the plasma membrane. FM4-64 is widely used as a non-specific marker of endocytosis and vesicle trafficking in living cells (Fischer-Parton et al., 2000). These observations suggest that AtBro1 is involved in the regulation of plasma-membrane homeostasis in Arabidopsis cells.

### AtBro1 regulates the response to ABA and mannitol stress

To determine the biological function of AtBro1 in seedling development, we studied a T-DNA insertion mutant of AtBro1, *bro1-1* (SALK_204462C), which has T-DNA inserted into intron six as shown in **Figure 1A**. After selection of a homozygous line of the *bro1-1* mutant, semi-quantitative RT-PCR and quantitative RT-PCR (qRT-PCR) were used to confirm the absence of AtBro1 transcripts in homozygous plants treated with and without ABA (**Figures 1B–D** right), as AtBro1 is highly induced by ABA (**Figure 1D** left). In addition, we generated AtBro1-overexpression lines (OXs) under the control of a CaMV 35S promoter in a WT Col-0 background. We selected three independent T1 OX lines, confirming overexpression by qRT-PCR (**Figure 1D** right and **Supporting Information Figure S1**).

**Figure 6:**
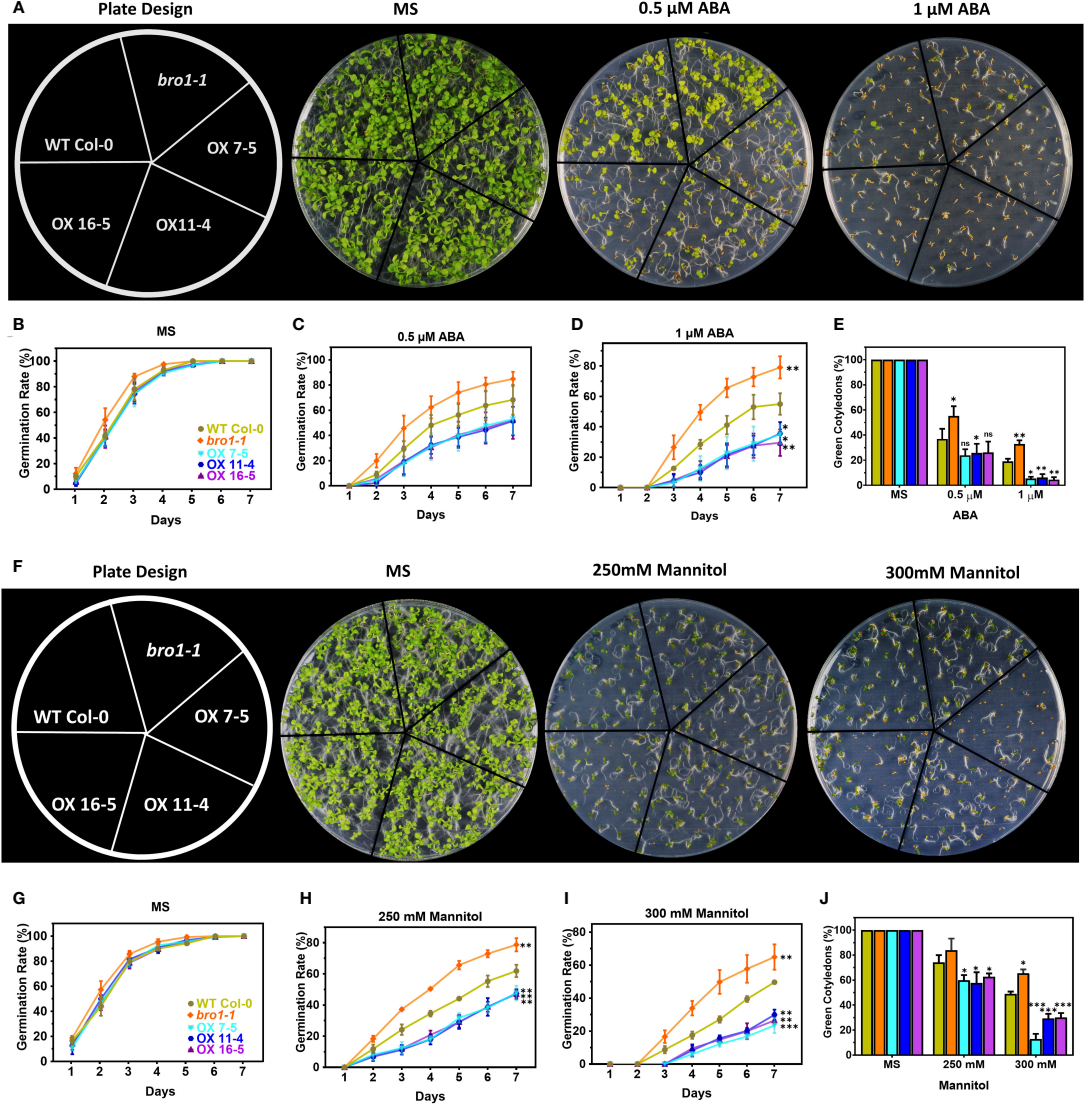
Response of *bro1-1* mutant and ATBro1-overexpressing lines to ABA and mannitol during seed germination. **(A, F)** Seed germination phenotypes. Vernalized seeds of WT Col-0, *bro1-1*, and AtBro1-OX lines (OX7-5, OX11-4, OX16-5) were sown on MS medium supplemented with 0.5 μM ABA, 1 μM ABA, 250 mM mannitol or 300 mM mannitol and grown for 9 days before plate images were recorded. Seed germination rates for ABA **(B–D)** and mannitol **(G–I)** treatments were calculated for the indicated time points. A minimum of 40 seeds per genotype were analysed in each replicate. Seeds from independent lines were used for replicates. Cotyledon greening ratios for ABA **(E)** and mannitol **(J)** treatments were calculated on day 10 after vernalisation. All data are means of three biological replicates ± SD. Significant differences were determined by Student *t*-test: ∗∗∗P < 0.001, ∗∗P < 0.01, ∗P < 0.05; ns, non-significant.

To elucidate the role of AtBro1 in stress responses, selected WT Col-0, *bro1-1*, OX7-5, OX11-4 and OX16-5 lines were grown with different concentrations of ABA or mannitol. The percentage germination was then calculated every day over seven days. No significant difference was observed between genotypes on MS medium without ABA or mannitol. However, germination of the *bro1-1* mutant was less sensitive than WT on medium with ABA or mannitol, whereas the OX transgenic lines were more sensitive to both treatments (**Figures 6A, F**). In response to ABA and mannitol treatment, the seed germination rate was lower in OX seeds than WT, although the *bro1-1* mutant showed more resistance to ABA inhibition than WT (**Figures 6B–D, G–I**). The percentage of green cotyledons in the above genotypes (**Figures 6E, J**) followed a similar pattern to the seed germination data, with the *bro1-1* mutant showing a higher proportion, and the OX lines a lower proportion than WT in the presence of either ABA or mannitol. To confirm the function of AtBro1 in these stress responses, we produced complementary lines (Comp-1 and Comp-2) obtained by crossing the *bro1-1* knockout plant with an OX line showing similar AtBro1 expression levels to WT plants. The phenotypes of the complementary lines were almost the same as those of the WT seedlings under ABA- and mannitol-stress conditions (**Figure S2**). These results led us to conclude that AtBro1 is involved in the response to ABA and mannitol during germination.

### *AtBro1* regulates the response to salt stress

**Figure 7:**
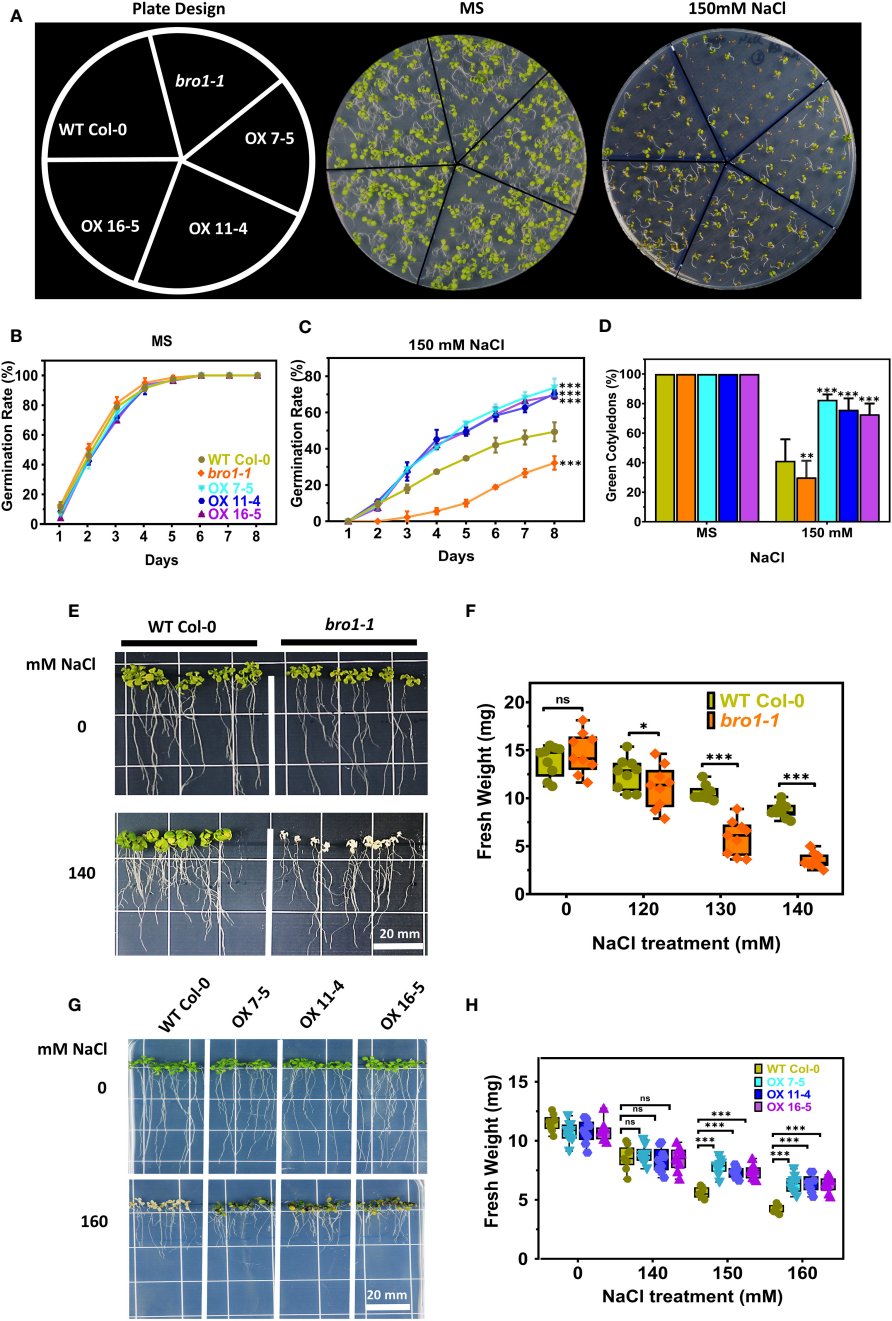
Salt-stress response of *bro1-1* and AtBro1-OX lines. **(A)** Seed germination rates were quantified daily from day 1 to day 7 after sowing for the indicated genotypes grown on MS medium with or without 150 mM NaCl. The percentages of cotyledon greening were recorded on the 9th day after sowing. At least 40 seeds per genotype in each replicate were used in three independent experiments. Values are mean ± SD of three replications. **(B, C)** Salt regulates germination rate of *bro1-1* and AtBro1-OX lines. **(D)** Ratio of green cotyledons on the 9th day after the end of the stratification period. All data are mean values of three biological replicates ± SE. Significant differences were determined by Student’s *t-*test: ∗∗∗P < 0.001, ∗∗P < 0.01, ∗P < 0.05; ns, non-significant. **(E–H)** Effect of salt-stress shock on plant survival. **(E, F)** Four-days-old seedlings (at least 30 seedlings for each experiment of biological replicate) of *A. thaliana* WT (Col-0) and *AtBro1-* knockout mutant (*bro1-1*) grown on MS medium were transferred to MS medium supplemented with various NaCl concentrations (120, 130 and 140 mM) and fresh weights were recorded after two weeks. **(G, H)** WT and AtBro1-OX lines (OX7-5, OX11-4, OX16-5) grown on MS medium were transferred to MS medium plus NaCl (140, 150 and 160 mM) and fresh weights were recorded after two weeks. Images were recorded 7 days after the transfer. Values are means ± SD (n = 3); **P < 0.01 (Student’s *t-*test). Scale bars = 20 mm.

We next tested whether AtBro1 affects the germination of seeds during salt stress. In the presence of NaCl, germination and the percentage of green cotyledons in the *bro1-1* mutant were both lower than in WT (**Figure 7A**), while in contrast germination rates (**Figures 7B, C**) and cotyledon greening ratios (**Figure 7D**) were much higher in the OX7-5, OX11-4 and OX16-5 transgenic lines. However, the *bro1-1* phenotype was restored to that of WT in the complementary lines Comp-1 and Comp-2 (**Figure S3**).

We performed a salt stress growth assay to further investigate the function of AtBro1 in NaCl tolerance. Seeds of WT Col-0, *bro1-1*, OX7-5, OX11-4 and OX16-5 were sown on MS medium. After four days, the seedlings were transferred to medium with different salt concentrations and allowed to grow vertically for a further eight days, when the fresh weights of all plants were determined. No difference was observed between WT Col-0, *bro1-1*, OX7-5, OX11-4 and OX16-5 seedlings under normal growth conditions on MS medium without salt. However, the *bro1-1* mutant showed a hypersensitive phenotype compared to WT when subjected to salt stress. In contrast, the OX7-5, OX11-4 and OX16-5 overexpression lines showed a significantly resistant phenotype (**Figures 7E, G**). This sensitivity profile was reflected in the fresh weights, with salt stress reducing the fresh weight of *bro1-1* mutant seedlings compared to WT (**Figure 7F**), while the OX lines showed higher weights (**Figure 7H**). Thus, AtBro1 is likely involved in the response to salt stress during germination.

### *AtBro1* regulates drought-stress resistance in soil

**Figure 8:**
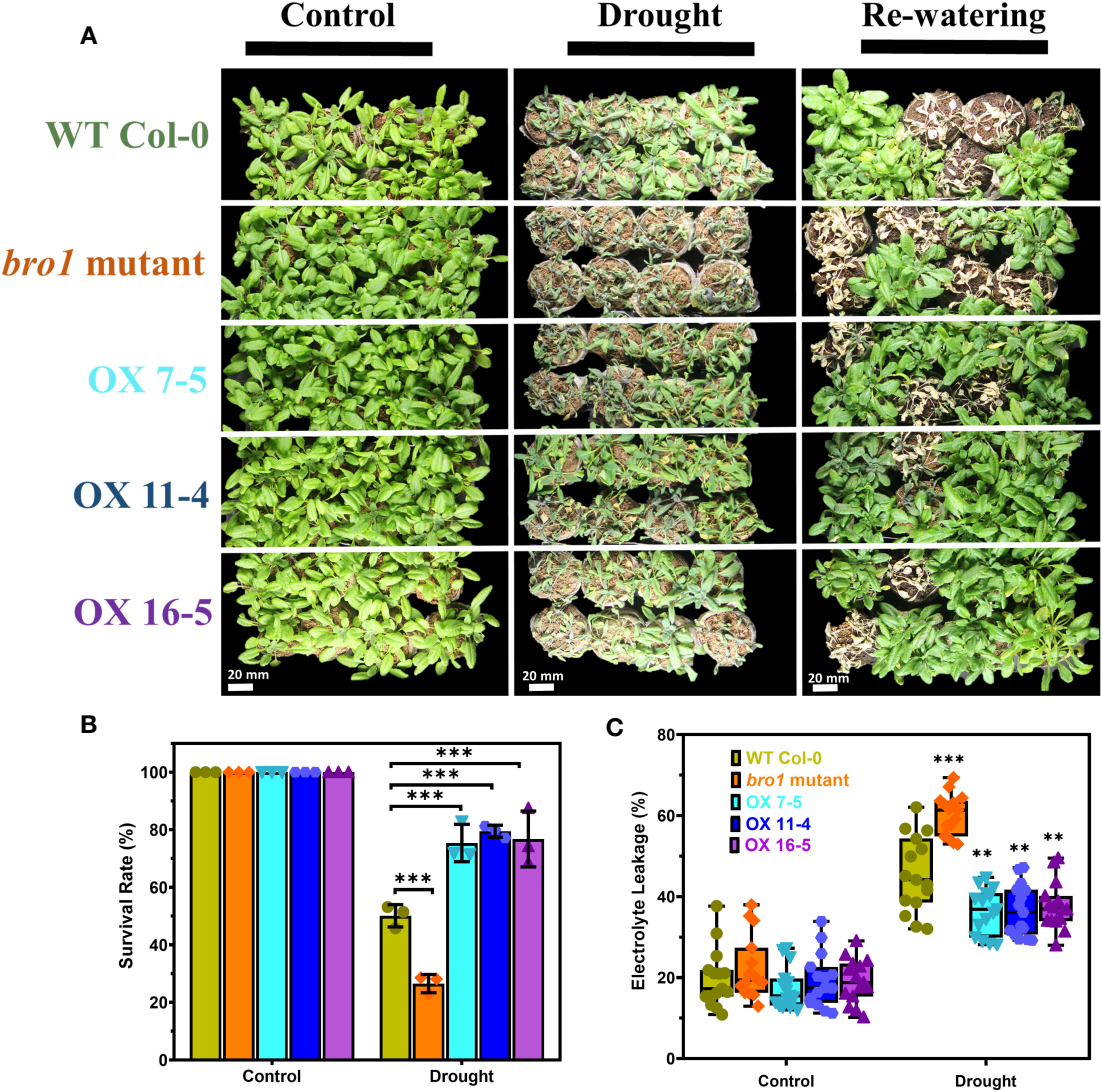
Enhanced tolerance of AtBro1-OX plants to drought stress. **(A)** The drought-tolerant phenotype of AtBro1-OX plants. Three-week-old WT Col-0, *bro1-1*, and AtBro1-OX plants were subjected to drought stress by withholding water for 10 days and then resuming watering for two days. Representative images were taken before (left panels) and after (middle panels) drought and after 4]four days of re-watering (right panels). **(B, C)** Electrolyte leakage and survival rates after re-watering. Data represent the mean ± standard error of three biological replicates, each evaluating 32 plants. Significant differences were determined by Student’s *t*-test: ∗∗∗*P* < 0.001, ∗∗*P* < 0.01. Scale bars = 20 mm.

We then turned to drought stress and sowed seeds of WT Col-0 and *bro1-1*, OX7-5, OX11-4 and OX16-5 plants in soil in a plant growth chamber. Under control conditions, there were no significant phenotypic differences in growth between the various genotypes. However, in the drought assay, the *bro1-1* mutant was more sensitive, as shown in **Figure 8A**, whereas the OX lines were more drought resistant compared to WT. Indeed, the highest survival rate after rewatering was observed in OX transgenic lines (**Figure 8B**). Ion leakage was measured to quantify stress damage after drought. Electrolyte leakage was significantly higher in *bro1-1* mutant plants subjected to drought, while leakage was lower in OX lines, than in WT (**Figure 8C**). These results confirm that AtBro1 plays a regulatory role in drought resistance.

### *AtBro1* affects the expression of signaling pathways and biosynthesis-related genes

**Figure 9:**
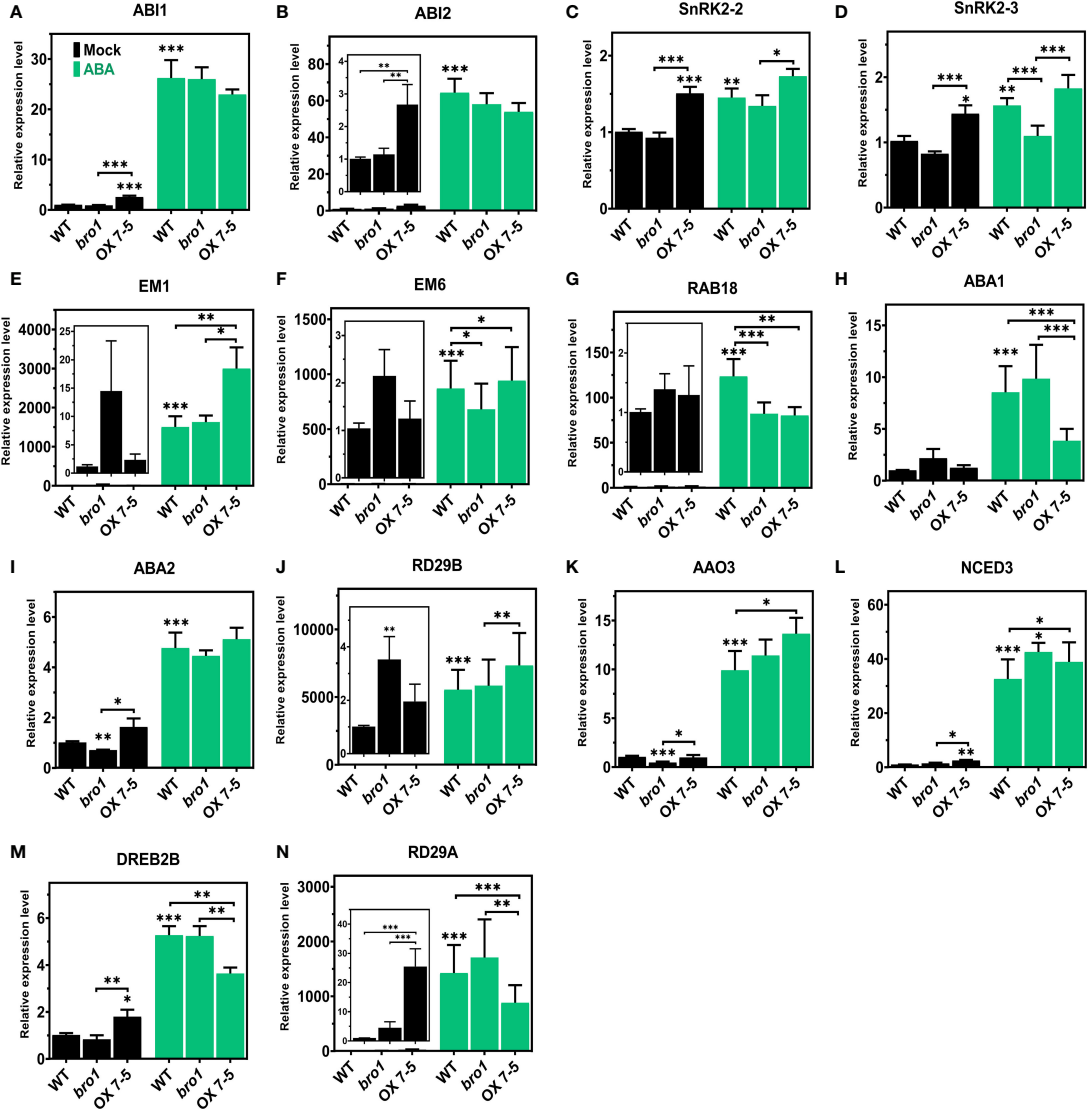
Expression levels of ABA signaling, ABA biosynthesis and ABA-independent genes in WT Col-0, *bro1-1* and OX 7-5 lines. Seeds of WT Col-0, *bro1-1* and OX 7-5 lines were germinated for two weeks and treated with or without 50 μM ABA for 4 h, then RNA was extracted and transcript levels of the indicated ABA signalling **(A–G)**, ABA biosynthetic **(H–L)** and ABA-independent genes **(M, N)** were analyzed by qRT-PCR. Values plus standard errors of the mean (n=3 biological replicates with independent technical triplicates) are shown. Significant differences were determined by Student’s *t*-test: ∗∗∗P < 0.001, ∗∗P < 0.01, ∗P < 0.05.

Our next step was to investigate the function of AtBro1 in ABA signalling. We isolated RNA from two-week-old seedlings of WT, *bro1-1* and OX 7-5 lines with or without 50 μM ABA treatment for 4 h, then checked the expression of a panel of genes involved in ABA signalling and ABA biosynthesis, as well as some ABA-independent genes. These included ABI1, ABI2, SnRK2.2, SnRK2.3, EARLYMETHIONINE-LABELLED 1 (EM1), EM6, RAB18, ABA1, ABA2, RD29A, RD29B, AAO3, NCED3 and DREB2B. The expression levels of the ABI1, ABI2 and SnRK2.2 genes were not affected by AtBro1, but SnRK2.3 was significantly upregulated in OX7 compared to WT, and downregulated in the *bro1-1* mutant line, after ABA treatment (**Figures 9A–D**). Genes related to the ABA signalling pathway, such as EM1 and EM6, were significantly upregulated in seedlings of AtBro1-OX transgenic lines, whereas they were significantly downregulated in *bro1-1* mutant seedlings (**Figures 9E, F**). Intriguingly, RAB18 was downregulated (**Figure 9G**), while other ABA biosynthesis genes (ABA2, RD29B, AAO3 and NCED3) were upregulated in both *bro1-1* and OX lines after ABA treatment; ABA1 was upregulated in the *bro1-1* mutant and downregulated in OX7-5 seedlings (**Figures 9H–L**). We also observed that two ABA-independent genes, DREB2B and RD29A, were downregulated in OX7-5 seedlings compared to the *bro1-1* mutant (**Figure 9M, N**). Taken together, these results indicated that AtBro1 directly or indirectly targets genes involved in ABA biosynthesis and signalling in Arabidopsis.

### AtBro1-regulated genes revealed by global RNA-seq analyses

**Figure 10:**
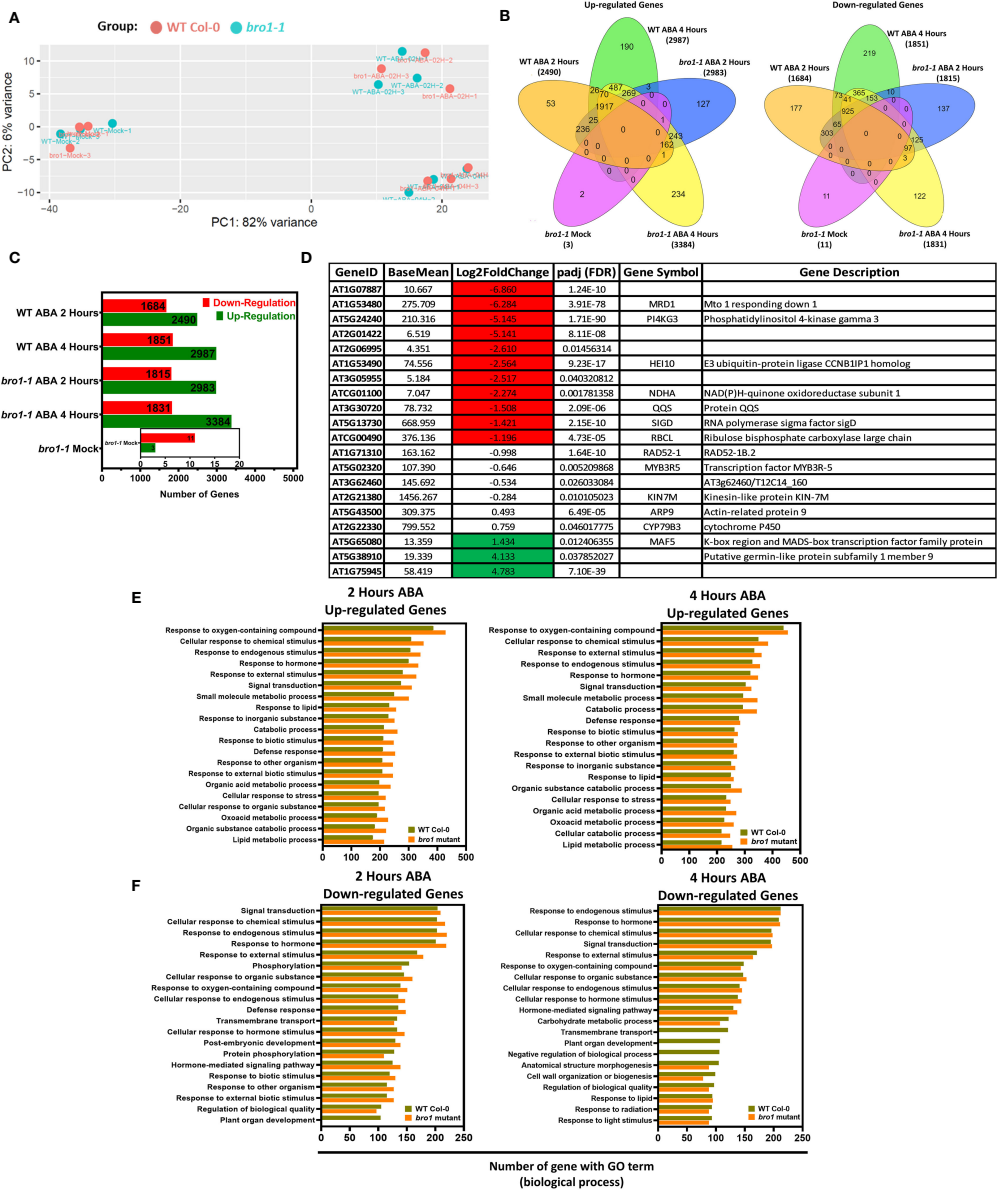
Comparative transcriptomes of *bro1-1* mutant and WT Col-0 plants with or without ABA treatment for 2 and 4 hours. **(A)** Principal component analysis of normalized transcriptome data. **(B)** Venn diagrams showing up- and downregulated genes in the *bro1-1* mutant compared to WT Col-0. **(C)** Number of DEGs in *bro1-1* vs WT Col-0 with or without ABA treatment for 2 and 4 h. **(D)** All gene patterns up- (highlighted green) and downregulated (highlighted red) in the *bro1-1* mutant compared to the WT Col-0 in control conditions. **(E, F)** GO terms that are significantly enriched among upregulated **(E)** and downregulated genes **(F)** in ABA-treated *bro1-1* compared to WT Col-0 plants (FDR < 0.05).

After documenting the involvement of AtBro1 in the response of Arabidopsis to several abiotic stresses, we were curious to uncover the regulatory network underpinning its activity. We therefore decided to perform RNA-seq analysis to explore the set of genes that were differentially expressed (DEGs) (fold change more than 1.0 and FDR < 0.05) between two-week-old WT and *bro1-1* mutant seedlings after treatment with ABA for 0, 2 and 4 h. Several genes were scattered shown as blue dots and most of the genes were concentrated as shown in **Supporting Information Figure S4**, indicating that AtBro1 has a strongly effect on the expression of these genes. To infer the systematic patterns of six variations in the data, principal component analysis (PCA) was performed to group the expression profiles in the experiment of each sample. The PCA showed that control (mock) samples and the expression profiles resulting from 2 and 4 h ABA treatment were apparently closely related groups in both WT and the *bro1-1* mutant (**Figure 10A**). Comparative analysis showed that the number of DEGs was higher between *bro1-1* treated with ABA and WT treated with ABA (both 2 h and 4 h treament groups), showing that AtBro1 modulates expression profiles in the presence of ABA (**Figure 10B**). In addition, a few genes were also differentially expressed between untreated *bro1-1* and WT seedlings. Genes that showed significant changes in expression were selected for further analysis, and this resulted in a greater number of upregulated genes in the *bro1-1* mutant after 2 (2983 genes) and 4 h (3384 genes) ABA treatment than in WT after 2 (2590 genes) and 4 h (2987 genes) ABA treatment, whereas the number of downregulated genes was almost the same in both genotypes with or without ABA treatment (**Figure 10C** and **Supporting Information Tables S2**–**5**).

In addition, we also performed an analysis to observe the transcript levels of DEGs in the *bro1-1* mutant vs. WT Col-0 (both mock-treated). As shown in **Figure 10D**, the expression of some genes, such as MRD1, PI4KGamma3, HEI10, NDHA, QQS, SIGD and RBCL, was significantly lower in the *bro1-1* mock sample vs the WT Col-0 mock samples. Transcript levels of some genes related to putative germin-like protein subfamily 1 member 9 and MAF5 were also higher in *bro1-1* mock-treated plants than in WT Col-0 mock plants. Moreover, AtBro1 also influenced the expression of several genes associated with other Arabidopsis processes (**Figure 10D**).

To explore the regulatory network of AtBro1 in response to short- and long-term ABA treatment, we also performed gene ontology (GO) and KEGG analysis on the set of significantly up- and downregulated genes: *bro1-1* mock vs. WT mock and *bro1-1* with ABA vs. WT with ABA, for the 2- and 4-h pairwise groups. Among the upregulated genes, those involved in the response to chemical stimulus, hormone, endogenous stimulus, external stimulus and defence response were highly enriched (**Figure 10E** and **Supporting Information Tables S6**–**9**). Among the downregulated genes, several categories, including chemical, hormonal, phosphorylation and external stimulus response, showed preferential enrichment in (*bro1-1* vs. WT)-ABA for 2 h or 4 h (**Figure 10F** and **Supporting Information Tables S6** **9**). In the presence of ABA, various downregulated genes from the comparison of *bro1-1* with ABA vs. WT with ABA for 2 or 4 h and that were assigned terms relating to plant organ development and transmembrane transport were less enriched in *bro1-1* than in WT Col-0 (**Figure 10F**). Together, the GO analysis suggests that the role of AtBro1 in abiotic tolerance involves regulation of ABA-responsive genes at the transcriptional level.

### Validation of DEG expression by qRT-PCR

**Figure 11:**
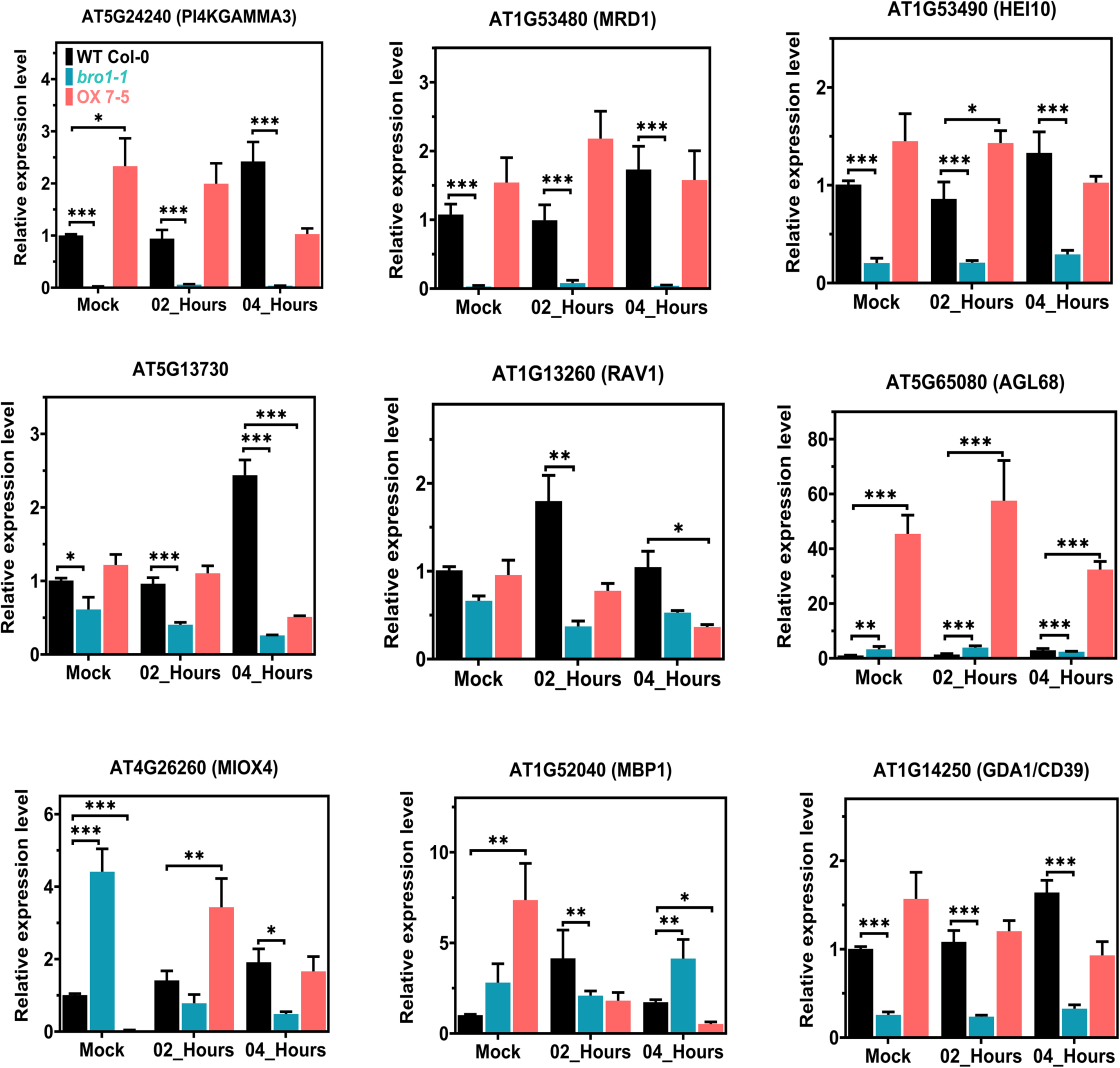
Transcript-level validation of RNA-seq analysis for ABA-responsive genes. Validation of the RNA-seq results by qRT–PCR analysis in WT Col-0, *bro1-1* and OX 7-5 lines. Seeds of WT Col-0, *bro1-1* and OX 7-5 lines were germinated on MS medium for two weeks and treated with or without 100 μM ABA for 2 and 4 h. Then RNA was extracted and selected genes were analyzed by qRT-PCR. Values plus standard errors of the mean (n=3 biological replicates with independent technical triplicates) are shown. Significant differences were determined by unpaired Student’s *t*-test: ∗∗∗P < 0.001, ∗∗P < 0.01, ∗P < 0.05.

We selected several genes shown to be up- or downregulated by the RNA-Seq data for validation by qRT-PCR. Since RNA-Seq was only performed in the WT and *bro1-1* mutant lines, we considered that if the selected genes were up- or downregulated in the *bro1-1* mutant compared to WT (with or without ABA), then these genes were likely to be down- or upregulated (i.e. to show the opposite expression profile) in the OX lines. To test this, we selected several genes such as AT5G24240 (PI4KGAMMA3/MOP9. 5), AT1G53480 (MRD1), AT1G53490 (HEI10), AT5G12730, AT1G13260 (RAV1), AT5G65080 (AGL68), AT4G26260 (MIOX4), AT1G52040 (MBP1) and AT1G14250 (GDA1/CD39) from our WT and *bro1-1* mutant (with or without ABA) data. We then examined the transcript levels of the selected candidate genes in WT Col-0, *bro1-1* and OX7-5 seedlings with or without ABA treatment by qRT-PCR (**Figure 11**).

The expression of PI4KGAMMA3/MOP9.5, MRD1, HEI10, AT5G12730, RAV1 and GDA1/CD39 was significantly lower in *bro1-1* than in WT. Interestingly, as we hypothesised, the expression of the majority of these genes, including PI4KGAMMA3/MOP9.5, MRD1, HEI10, AT5G12730 and GDA1/CD39, was upregulated in the OX7-5 line (**Figure 11**). However, the transcript levels of AGL68 and MBP1, which were clearly higher in *bro1-1* than in WT, were even more abundant in the OX7-5 line, as shown by the qRT-PCR results. Moreover, MIOX4 gene expression was higher in *bro1-1* than in the WT and OX lines without ABA, but was significantly lower in the *bro1-1* mutant line after 2 and 4 h ABA treatment compared to the WT and OX 7-5 lines. During ABA treatment, MIOX4 expression was upregulated in OX line compared to both WT and the *bro1-1* mutant after 2 h ABA treatment. Therefore, these co-regulated genes in the WT, *bro1-1* mutant and OX lines may directly or indirectly regulate the *AtBro1* gene. Overall, these results are consistent with the RNA-Seq results in WT Col-0 and the *bro1-1* mutant with or without ABA treatment.

## Discussion

Many physiological mechanisms in plants, such as the response to abiotic stress, seed dormancy and vegetative growth, are regulated by ABA (Cohen and Bray, 1990; Giraudat et al., 1994; Savouré et al., 1997; Gosti et al., 1999; Hoth et al., 2002; Yoshida et al., 2014; Sah et al., 2016). In our current study, we selected a gene responsive to ABA and abiotic stress conditions, *AtBro1*, which encodes a Bro1-like domain-containing protein in Arabidopsis. We investigated the role of *AtBro1* in the plant response to abiotic stress by physiological, biochemical and genetic characterisation of *bro1-1*, OX7-5, OX11-4 and OX16-5 plants. Our focus was on gaining a better understanding of the functional processes governed by AtBro1 during abiotic stress. In addition, we identified several antagonistic phenotypes of AtBro1-OX lines. To date, the various biological roles of the AtBro1 gene and its homologues in other plant species (**Figures 2B, C**) remain a mystery.

A Bro1-domain-containing protein has been reported to play a role in yeast, where cell proliferation is impaired in a *brol* mutant in the absence of sufficient nutrients, leading to decreased cell viability (Nickas and Yaffe, 1996; Odorizzi et al., 2003). In Arabidopsis, the plant Bro1 protein BRAF regulates FREE1 and vacuolar degradation of membrane cargo (Shen et al., 2016) and the deubiquitinating enzyme AMSH3 is regulated by ALIX (Kalinowska et al., 2015). In addition, a mutation in the Bro1 domain leads to altered vacuole morphogenesis (Cardona-López et al., 2015). In the current study, we found AtBro1 protein to be localized to the plasma membrane of the cell (**Figure 5**). Similarly, the AtOZF1 (Huang et al., 2011) and AtOZF2 (Huang et al., 2012) proteins localize to the plasma membrane and are also involved in the response to various abiotic stresses. Moreover, signaling events occur at the plasma membrane following extracellular ABA perception (Hubbard et al., 2010; Rodriguez et al., 2014) and membrane proteins involved in stress responses have putative functions in the repair and protection of membranes (Malakshah et al., 2007), as well as signal transduction (Osakabe et al., 2010). Several of our results also support the hypothesis that AtBro1 could be involved in the abiotic stress response through an ABA-dependent signal transduction pathway.

The abundance of *AtBro1* transcripts significantly increased in the presence of ABA, sodium chloride and mannitol (**Figure 3**), peaking after 2 h in all three cases, consistent with the notion that AtBro1 is involved in the response to multiple abiotic stresses. Furthermore, when driven by the *AtBro1* promoter, the GUS expression pattern (**Figure 4D**) reflects the qPCR results, showing expression in rosette leaves and floral clusters, especially in anthers. This is reminiscent of the tissue-specific localisation of *NPC2* gene activity (Krčková et al., 2018): there is no expression in roots, as also found for some of the AtβCA family genes. For example, AtβCA2p was completely absent in roots while expression was higher in floral clusters (Wang et al., 2014); AtβCA2p is also reported to be involved in stress responses (Miyazaki et al., 2021). The same observations have also been reported for the *AtSRP2* and *AtSRP3* genes, which are highly expressed in floral clusters but not in roots (Ahn et al., 2009) and are involved in stress responses (Laibach et al., 2018). pAtBro1-GUS expression was more prominent in seedling leaves after ABA treatment.

We found that the *bro1-1* knockout mutant is less sensitive to mannitol stress, but more sensitive to NaCl stress, than WT, as evidenced by seed germination rates and the proportion of green cotyledons in seedlings. In contrast, OX plants are hypersensitive to mannitol (**Figure 6**), but show improved salt tolerance during seed germination and seedling growth (**Figure 7**). The *bro1-1* mutant also shows early seed germination and less inhibition by ABA of seed germination and postgermination growth. On the other hand, AtBro1-OX lines are hypersensitive to the effects of ABA on germination and seedling growth. In a report on the Bro1-domain-containing protein, ALIX, the seed germination and root growth rate of *alix-1* mutant plants were also affected by ABA (García-León et al., 2019). As we observed that AtBro1 overexpression plants were hypersensitive to mannitol stress during seed germination, and showed drought avoidance compared with the wild-type and *bro1-1* mutant plants. Thus, it appears that AtBro1 may be involved in ABA-regulated, rather than osmotic-stress, responses. This is similar to the case with AtAIRP4, as AtAIRP4-OX plants are hypersensitive to osmotic stress during seed germination, but more resistant to drought stress (Yang et al., 2016). Interestingly, the phenotypes we observed in the Arabidopsis *bro1-1* mutant and OX lines after ABA treatment are similar to those reported for monocots (Kawakatsu et al., 2009; Tian et al., 2015; Wang et al., 2020). Moreover, our results are consistent with a previous study on the *AGL21* and *ARR5* genes: in the presence of various concentrations of ABA, *agl21* mutant seeds are more resistant to mannitol and ABA inhibition, while AGL21-OX seeds are more sensitive to ABA and mannitol during germination (Yu et al., 2017). Similarly, ARR5-OX transgenic plants show hypersensitivity to ABA during germination, whereas an *arr5* loss-of-function mutant is insensitive to ABA; ARR5-OX transgenic plants also displayed enhanced drought tolerance after re-watering compared with WT (Huang et al., 2018). Thus, these results lead us to hypothesise that AtBro1 is involved in the plant response to ABA and mannitol stress conditions. Electrolyte leakage is a marker of cell membrane damage in plants (Bandurska and Gniazdowska-Skoczek, 1995; Gopi et al., 2007; Radoglou et al., 2007; Khair and Karim, 2015). Under normal growth conditions, the phenotype and membrane integrity of WT, *bro1-1* and OX lines are similar. However, when subjected to drought, the *bro1-1* mutant clearly performs less well than OX lines and WT plants. Indeed, the OX lines are less likely to wilt and show less electrolyte leakage than WT and the *bro1-1* mutant, and this is associated with an improved survival rate in the transgenic plants (**Figure 8**). These results suggest that AtBro1 plays an important role in maintaining cell membrane integrity, thereby reducing water loss during drought stress (Osakabe et al., 2010). Taken together, our data show that AtBro1 overexpression confers superior tolerance to drought.

We also found that many stress-response genes associated with ABA biosynthesis, ABA signalling and stress responses were upregulated following ABA treatment (**Figure 9**). Several stress-related genes, including SnRK2.3, NCED3, AAO3, and RD29B, are highly expressed in AtBro1-OX plants compared to WT Col-0, whereas ABA1 and RD29A expression decreased in OX lines. Changes in transcript levels are indicative of the degree of stress sensitivity or tolerance under harsh conditions (Wan and Li, 2006; Ebrahimian-Motlagh et al., 2017; Li et al., 2019). The increased drought tolerance observed in AtBro1-OX plants is likely controlled by different endogenously produced ABA levels and altered ABA responses. ABA biosynthesis is influenced by the induction of several genes, including *NCED3* and *AAO3* (Wan and Li, 2006; Vishal and Kumar, 2018). Our qRT-PCR experiments show that AtBro1 affects the transcript levels of various stress-related genes, such as *RD29B*, *SnRK2.3*, *AAO3* and *NCED3*. ABI1 and ABI2 are type 2C serine/threonine phosphatases that act as key regulators in the responses of Arabidopsis to ABA and salt stress (Merlot et al., 2001; Ohta et al., 2003). From our results, it seems likely that, under ABA-treatment conditions, AtBro1 exerts regulatory control upstream of such ABA- or stress-related genes.

To study the role of AtBro1 at the level of transcriptional regulation and gain insight into the possible biological mechanisms by which it exerts its function, analysis of RNA-sequencing data was performed. Transcriptome and Gene Ontology analysis of *bro1-1* versus WT Col-0 plants with or without ABA treatment revealed that AtBro1 affects various biological functions, especially the cellular response to ABA stimulus, the response to biotic stimulus, and the regulation of stress responses. GO analysis showed that numerous genes are significantly enriched in terms related to external stimulus, response to hormone, defence response and hormone-mediated signalling pathway. This indicates that the enhanced seed germination of the *bro1-1* mutant may be due to increased ion and osmotic signalling; this signalling can induce downstream stress-responsive genes, such as the genes that respond to ABA, as shown in **Figures 10B, C** and **Supporting Information Tables S2**–**5**. These genes are involved in abiotic stress responses and contribute to plant resistance to various stress factors when overexpressed (Golldack et al., 2014; Lim et al., 2022). This analysis confirms that ABA-regulated genes are involved in AtBro1-mediated abiotic stress tolerance.

A number of transcription factors, kinases and phosphatases are involved in stress-induced hormonal signalling and may also be involved in the regulatory mechanisms occurring in seeds (Oracz and Karpiński, 2016). E3 ligases are also implicated in hormone signalling (Kelley, 2018). Various abiotic stress-responsive genes were repressed or induced by ABA treatment according to the RNA-sequencing data (**Figure 10D**), including MOP9.5, MRD1, HEI10 and MIOX4, which have previously been shown to be regulated by abiotic stress or ABA (Akhter et al., 2016; Nepal et al., 2020; Kim et al., 2022; Singh et al., 2022). The AtPI4Kgamma3/MOP9.5 gene is involved in the regulation of Arabidopsis PtdI4 kinase and in the response to high salt conditions or ABA treatment. It is highly induced at early developmental stages and its expression is also high in the reproductive organs (Akhter et al., 2016). Furthermore, MOP9.5 expression increases specifically in a mutant of *AtCAF1b*, which is activated in response to abiotic stress (Walley et al., 2010). The MOP9.5 gene is hypomethylated and its transcript level is reduced in an EARLY FLOWERING IN SHORT DAYS (EFS) mutant (Cheng et al., 2018). In a previous study, it was reported that AtMRD1 expression is lower in both rosette and young silique tissues of a *mto1-1* knockout. MTO1-RESPONDING DOWN 1 (MRD1) is controlled by OZF1 at the transcriptional level in the response to salicylic acid and promotes defence in Arabidopsis (Singh et al., 2022). MRD1 is downregulated in *sdg4* mutant flowers and SDG4 is involved in the modulation of genes that have a role in pollen-tube growth (Cartagena et al., 2008). HEI10 is considered to be a ubiquitin or SUMO E3 ligase involved in the control of crossover formation in various eukaryotes (Pyatnitskaya et al., 2019). Strong interactions have been observed between HEI10 and other proteins involved in interference-sensitive meiotic crossovers and crossover positioning (Li et al., 2021; Morgan et al., 2021; Nageswaran et al., 2021; Durand et al., 2022). It is also thought that HEAT SHOCK FACTOR BINDING PROTEIN (HSBP) inhibits the expression of HEI10 (Kim et al., 2022). In our study, we observed that the represention of PI4KGAMMA3/MOP9.5, MRD1 and HEI10 in the transcriptome decreased in *bro1-1* mutant plants compared to WT, but was higher in OX lines during ABA treatment (**Figure 11**).

It has been reported that Arabidopsis lines overexpressing a myo-inositol oxygenase gene (MIOX4) show improved photosynthetic efficiency (Nepal et al., 2019) and are less affected by salt, cold and heat (Lisko et al., 2013; Nepal et al., 2020). MIOX4 expression, which severely disrupts normal plant growth and development, is induced in EsWAX1-OX lines (Zhu et al., 2014). Nevertheless, we observed that MIOX4 is highly induced in the AtBro1 mutant compared to WT and the AtBro1-OX lines, while it is expressed at lower levels in the *bro1-1* mutant line after 2 and 4 h ABA treatment (**Figure 11**), indicating that MOP9.5, MRD1, HEI10 and MIOX4, genes function directly or indirectly in the regulation of AtBro1 and also in the abiotic stress response in Arabidopsis. Further research is needed to fully elucidate the function of MOP9.5, MRD1, HEI10 and MIOX4 along with the *AtBro1* gene and to understand the regulatory interactions between these genes.

## Conclusion

**Figure 12:**
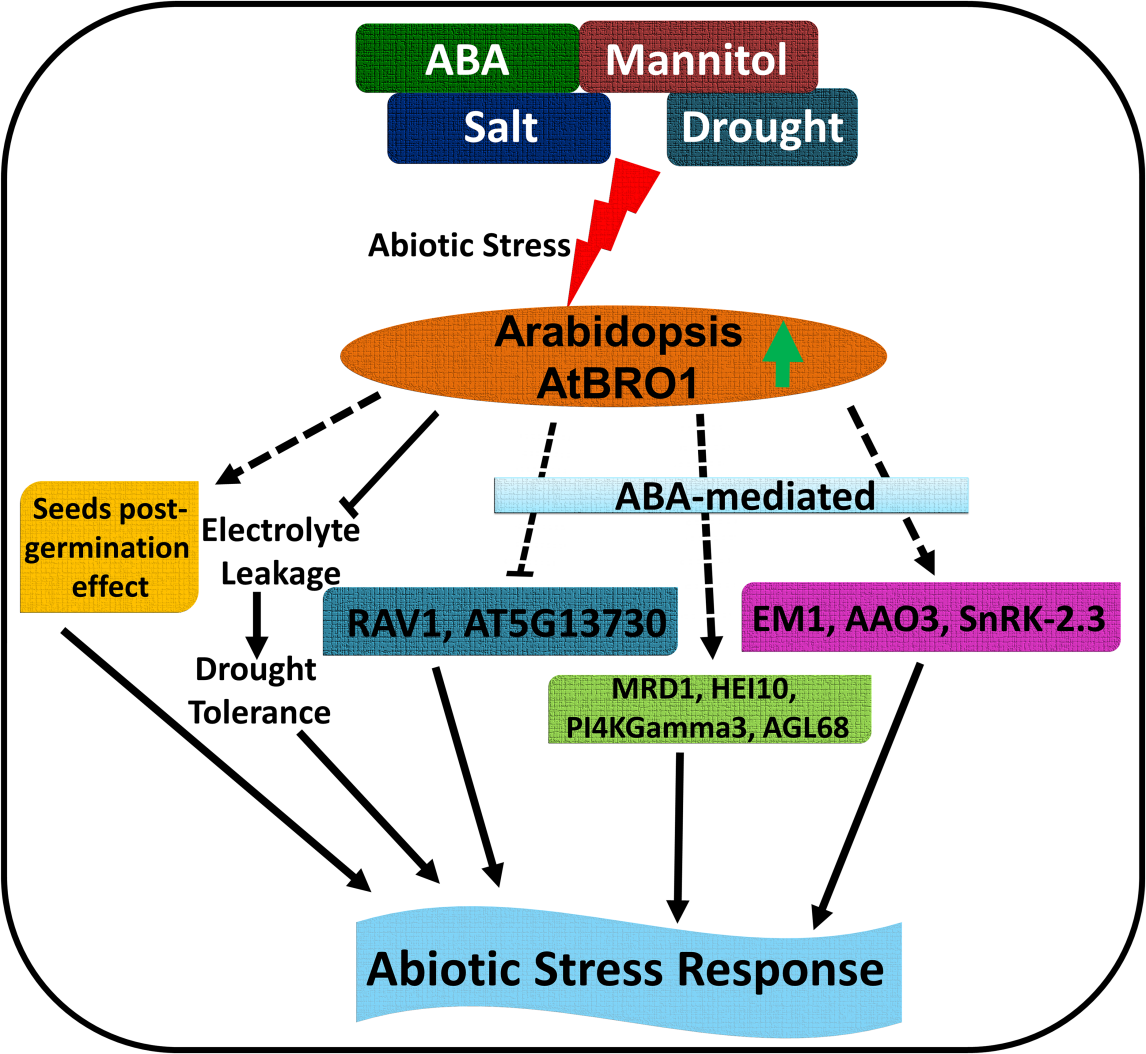
Schematic model of the role of AtBro1 in the plant response to abiotic stress. Green up-directed arrow shows upregulation of AtBro1 in response to abiotic stress. Blunt arrows (┴) indicate inhibition. Arrows at the end of solid lines indicate positive and direct or indirect regulation; arrows at the end of dashed lines indicate hypothetical or indirect regulation.

Plants can adapt to range of environments and respond to unfavourable conditions by distributing resources to cope with stress-related factors and by slowing or stopping growth. It is important to understand the molecular mechanisms underpinning these responses and, in our study, we have addressed the role of a crucial regulator, the plasma membrane-localised protein AtBro1, which is involved in the response to abiotic stress conditions when environmental stress factors are absent or act at low levels. And as we shown that *AtBRO1* has orthologs in different crop plants so it will be interesting to observe regulation of Bro1-domain containing protein in different other crops during abiotic stress. AtBro1 regulates plant stress tolerance by increasing or decreasing the expression of a large number of stress-responsive target genes, including MOP9.5, MRD1, HEI10 and MIOX4, to maintain ion homeostasis and other critical stress response elements (**Figure 12**). Our study suggests that AtBro1 is involved in balancing plant stress responses and growth, and is thus a promising candidate for interventions that improve plant stress resistance.

## Data availability statement

The original contributions presented in the study are included in the article/supplementary material, further inquiries can be directed to the corresponding author/s. The RNA-Seq data have been deposited for public use in the Gene Expression Omnibus (GEO) under accession number GSE225062. This data can be found here: https://www.ncbi.nlm.nih.gov/geo/query/acc.cgi?acc=GSE225062.

## Author contributions

Conceptualization, AL and SM. Methodology, SM, and MS. Validation, SM, MS and AL. Formal analysis, AL and SM. Investigation, SM, and MS. Data curation, SM and MS. Writing—original draft preparation, SM. Writing—review and editing, SM, MS and AL. Visualization, SM. Supervision, AL. Project administration AL. Funding acquisition, AL. All authors contributed to the article and approved the submitted version.
