# Supplementary material for: The Bro1-like domain-containing protein, AtBro1, modulates growth and abiotic stress responses in Arabidopsis
Source: Front Plant Sci. 2023 May 12;14:1157435. doi: 10.3389/fpls.2023.1157435 (PMC10213323; doi:10.3389/fpls.2023.1157435)

**Supporting Information**

**Figure S1.** Identification of AtBro1 overexpression transgenic lines.

**Figure S2:** Response of *bro1-1* mutant and complementation lines to abscisic acid (ABA) and mannitol in seed germination.

**Figure S3:** Salt stress response of *bro1-1* mutant and complementation lines (Comp-1 and 2) in seed germination.

**Figure S4.** MA-plots were generated for WT Col-0 and *bro1-1* mutant with mock, ABA 2-hour, and 4-hour group comparisons.

**Table S1.** Primers used in this study.

**Table S2.** List of assembled transcripts that were up-regulated or down-regulated (log2 fold change (log2FC) > 1 or <-1, false discovery rate (FDR ) < 0.05) in WT ABA 02 Hours vs Mock WT Col-0 Arabidopsis seedlings.

**Table S3.** List of assembled transcripts that were up-regulated or down-regulated (log2 fold change (log2FC) > 1 or <-1, false discovery rate (FDR ) < 0.05) in WT ABA 04 Hours vs Mock WT Col-0 Arabidopsis seedlings.

**Table S4.** List of assembled transcripts that were up-regulated or down-regulated (log2 fold change (log2FC) > 1 or <-1, false discovery rate (FDR ) < 0.05) in *bro1-1* mutant with ABA 02 Hours vs WT with ABA 02 Hours Seedlings

**Table S5.** List of assembled transcripts that were up-regulated or down-regulated (log2 fold change (log2FC) > 1 or <-1, false discovery rate (FDR ) < 0.05) in *bro1-1* mutant with ABA 04 Hours vs WT with ABA 04 Hours Seedlings

**Table S6.** Gene Ontology and KEGG pathway enrichment analysis of up-regulated or down-regulated genes in WT ABA 02 Hours vs Mock WT Col-0 Arabidopsis seedlings

**Table S7.** Gene Ontology and KEGG pathway enrichment analysis of up-regulated or down-regulated genes in WT ABA 04 Hours vs Mock WT Col-0 Arabidopsis seedlings

**Table S8.** Gene Ontology and KEGG pathway enrichment analysis of up-regulated or down-regulated genes in the *bro1-1* mutant with ABA 02 Hours vs WT with ABA 02 Hours Arabidopsis Seedlings.

**Table S9.** Gene Ontology and KEGG pathway enrichment analysis of up-regulated or down-regulated genes in the *bro1-1* mutant with ABA 04 Hours vs WT with ABA 04 Hours Arabidopsis Seedlings

**FIGURE S1**:

**Identification of AtBro1 overexpression transgenic lines.**

Quantitative RT-PCR (qRT-PCR) of ATBro1 transcripts in WT Col-0 and T2 transgenic overexpression lines of ATBro1 to select independent overexpression lines for further experimental analysis, and the actin2 gene was used as an internal control.


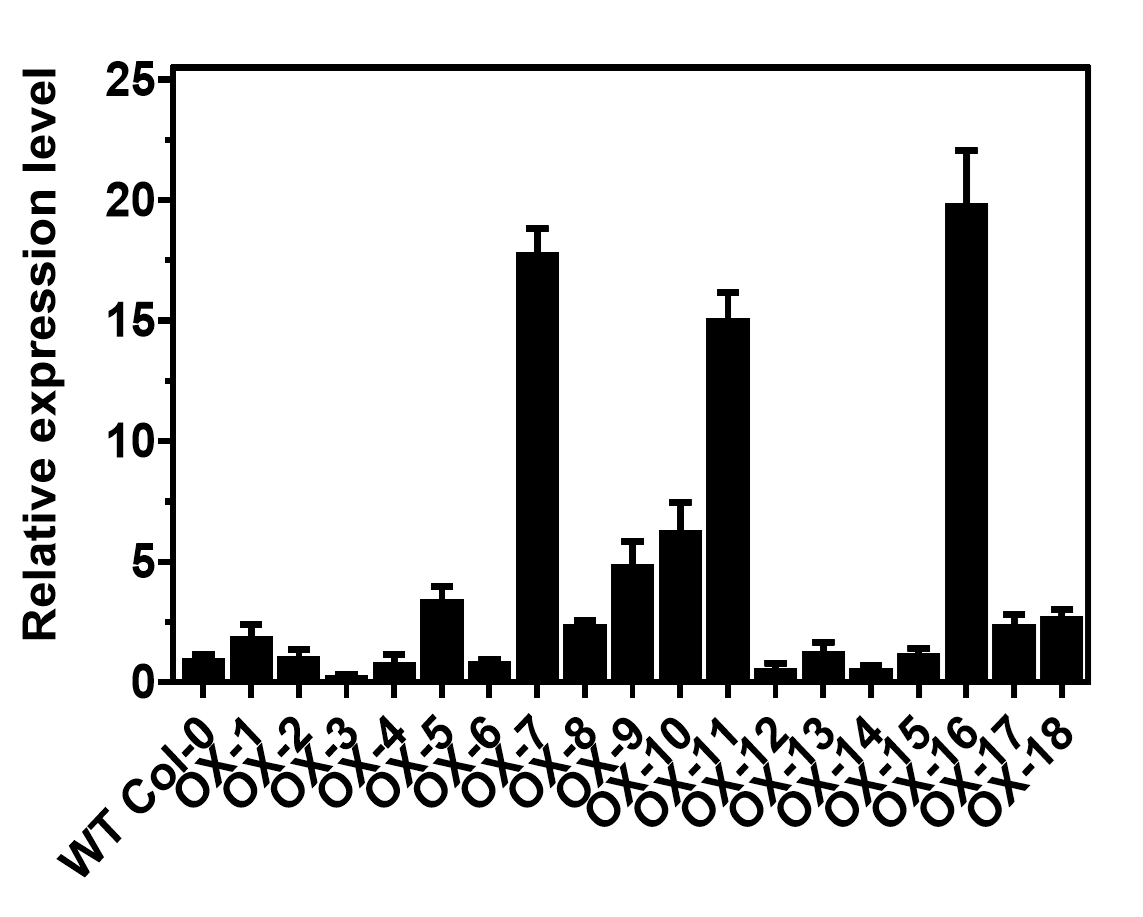


**FIGURE S2:**


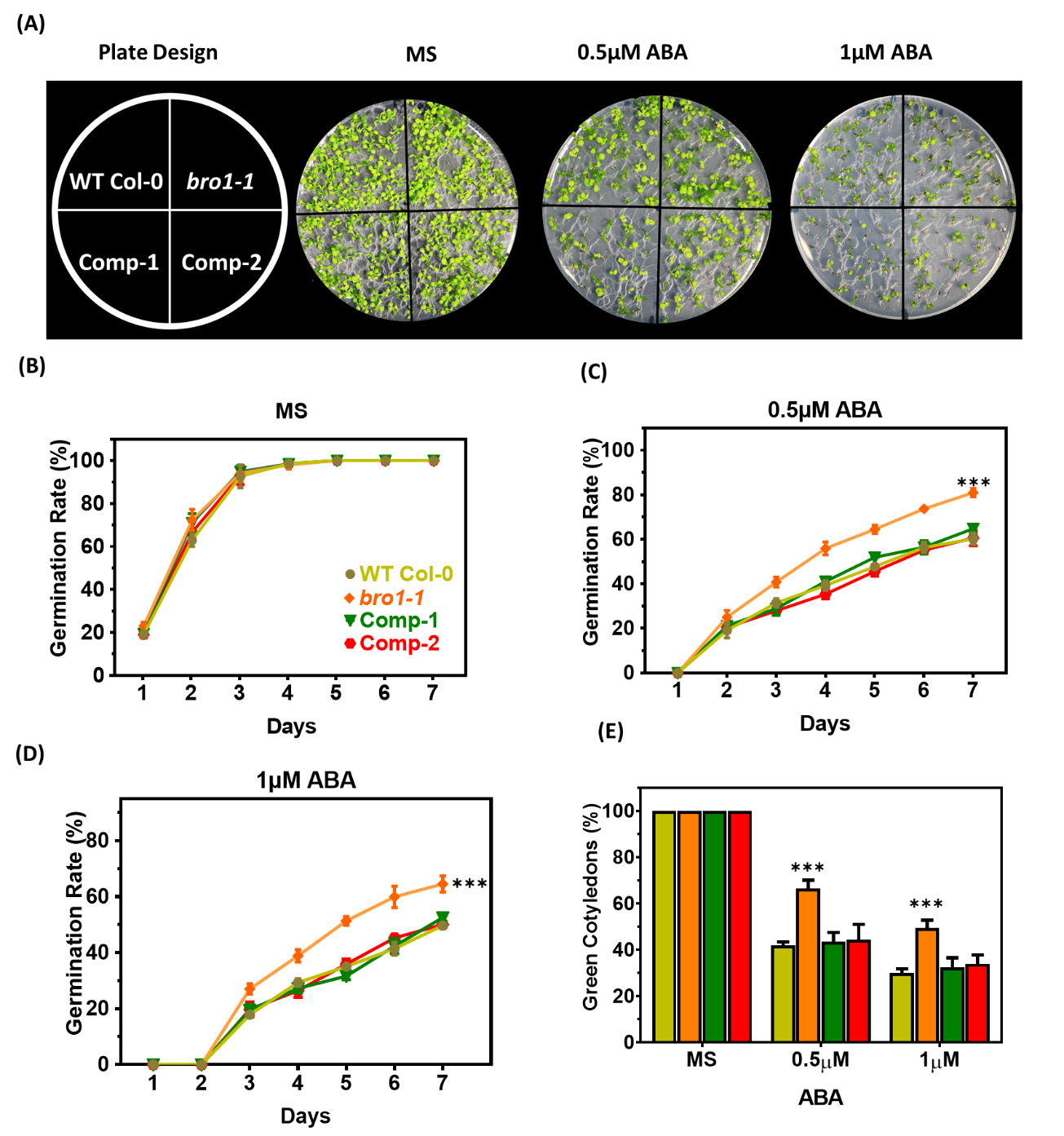
**Response of *bro1-1* mutant and complementation lines to abscisic acid (ABA) and mannitol in seed germination**. (A and F) Seed germination phenotypes. Vernalized seeds of Arabidopsis thaliana WT (Col-0), knockout mutant (*bro1-1*), and complementation lines (Comp-1 and 2) were sown on a Murashige & Skoog (MS) medium or MS medium containing 0.5 μM or 1 μM ABA and an MS medium or MS medium containing 250mM and 300 mM mannitol grown for 9 d before the images were recorded on 12^th^ day. Seed germination curves; germination rates for ABA treatment (B-D) and for mannitol (G-I) were calculated at the indicated time for the lines and treatments in (A) and (F). At least 50 seeds per genotype were measured in each replicate. Seeds from independent lines were used for replicates. Cotyledon greening ratio; green cotyledon ratios for ABA treatment (F) and for mannitol (J) were counted on Day 10 after the end of vernalization. All data are mean values of three biological replicates ± SE. The significant difference was determined by t-test: ∗P < 0.05, ∗∗P < 0.01, ∗∗∗P < 0.001, and ns=non-significant.


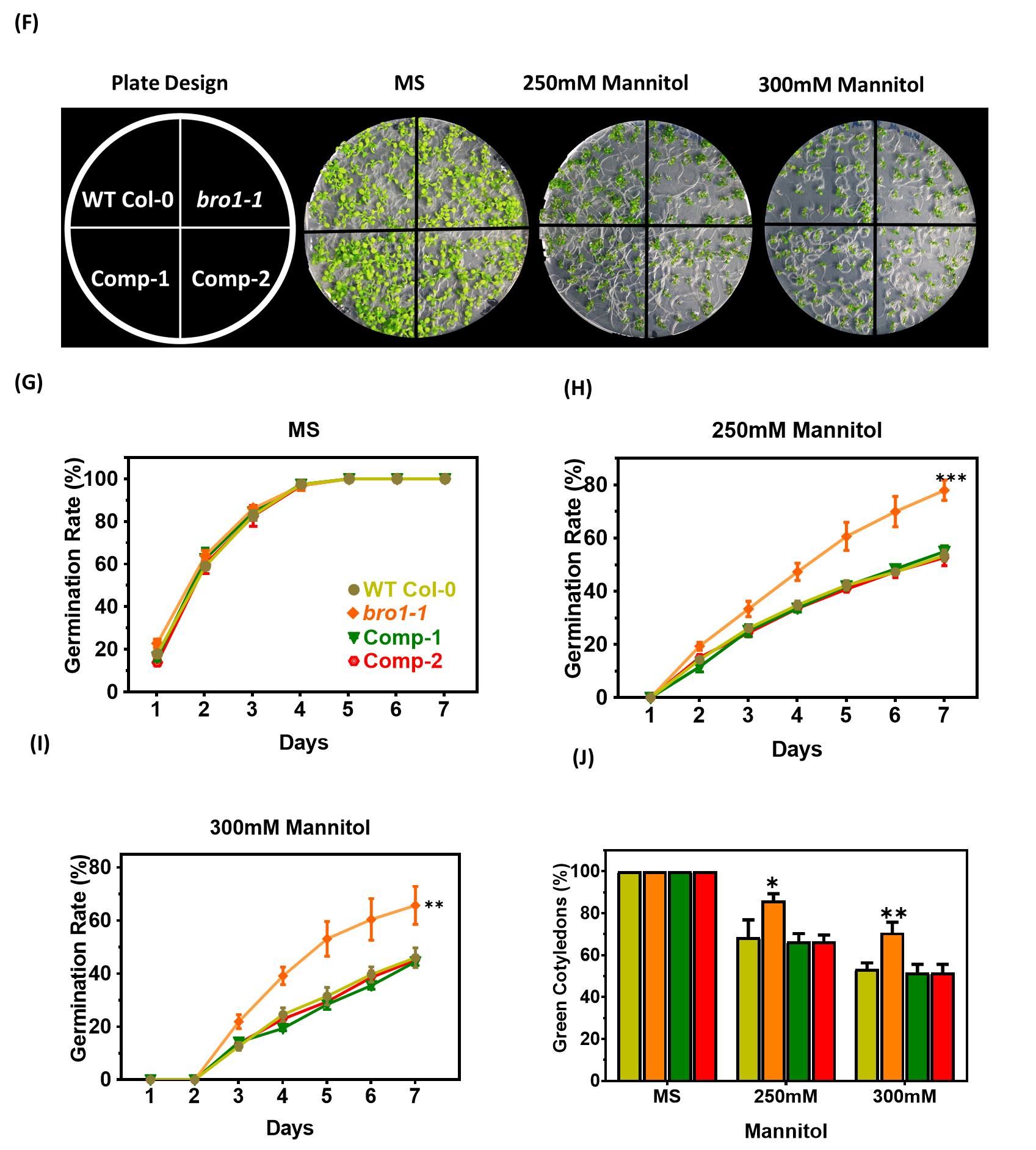


**FIGURE S3:**


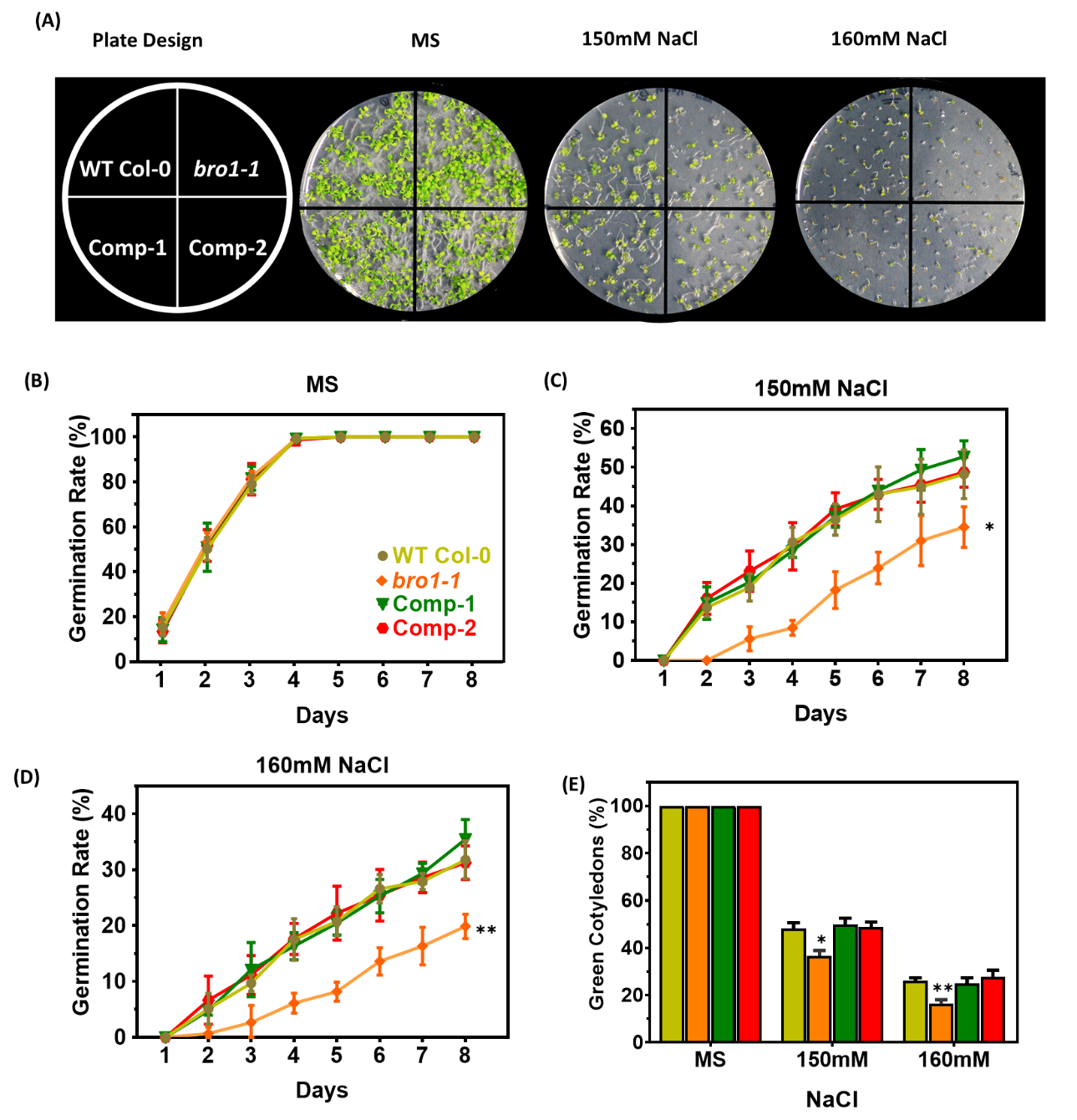
**Salt stress response of *bro1-1* mutant and complementation lines (Comp-1 and 2) in seed germination.** Seed germination percentages of the indicated genotypes grown on an MS medium or MS medium containing 150 and 160mM NaCl were quantified every day from the 1st day to the 7th day after sowing. Cotyledon-greening percentages were recorded on the 9th day. Three independent experiments were conducted, with at least 50 seeds per genotype in each replicate. Values are mean ± SD of three replications. (A) Photographs of seedlings grown on different media on day 8 after the end of stratification. (B–C) Seed germination rates of indicated genotypes grown on different media. (D) Green cotyledon ratio at day 9 after the end of stratification. All data are mean values of three biological replicates ± SE. The significant difference was determined by Student’s *t-test*: ∗P < 0.05, ∗∗P < 0.01, ∗∗∗P < 0.001, and ns=non-significant.

**FIGURE S4:**

**MA-plots were generated for WT Col-0 and *bro1-1* mutant with mock, ABA 2-hour, and 4-hour groups comparisons.** The MA-plots illustrate the distribution of the log fold change of the differentially expressed genes. Genes that are significantly differentially expressed are highlighted in blue, while those that are not, are displayed in grey. For each of the graphs the Y-axis shows the log fold change, and X-axis shows the mean normalized counts. Differentially expressed genes were determined using *DESeq2* (*False discovery rate*; *FDR* < 0.05).


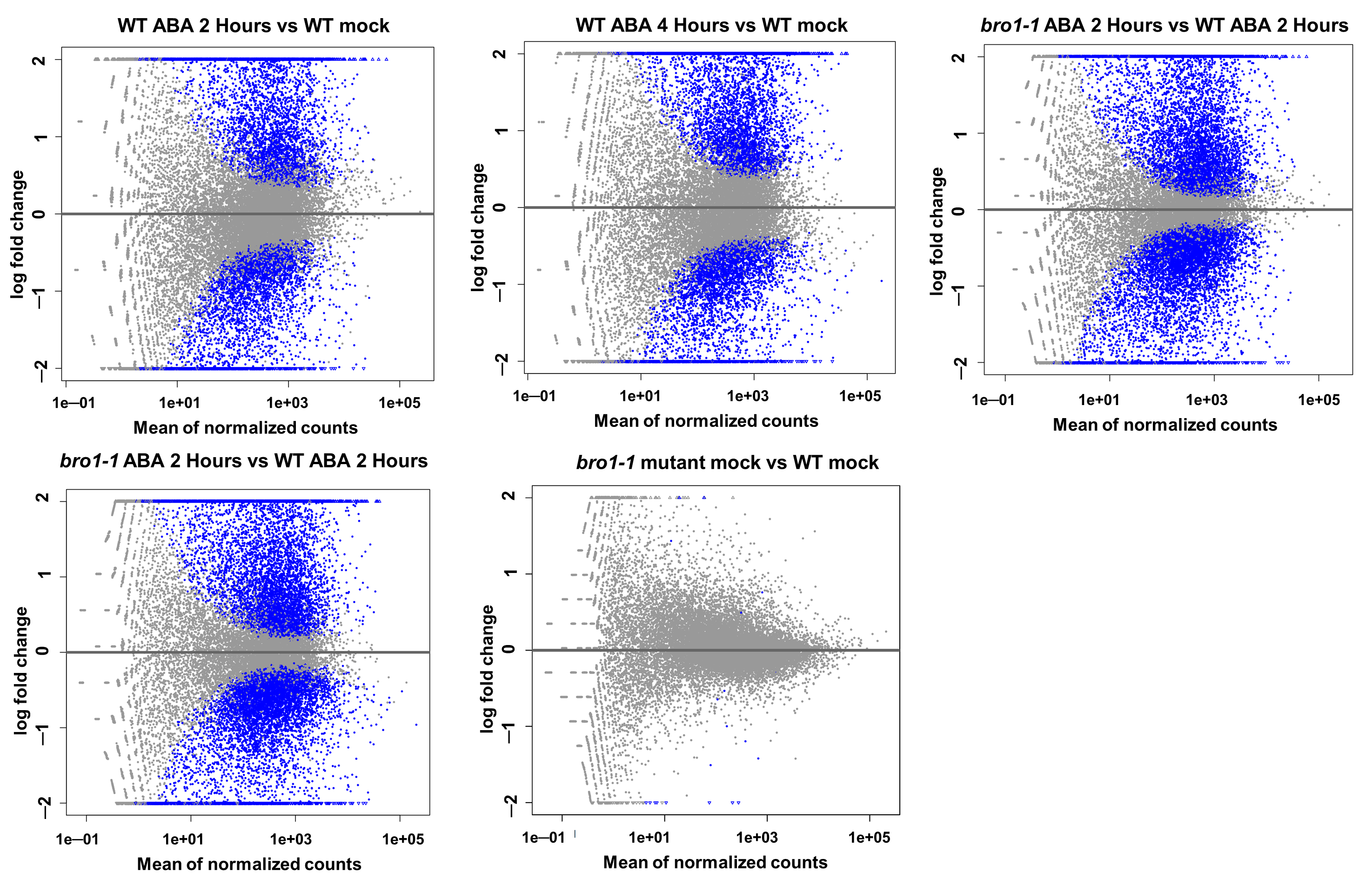

Supplement: Supplementary file 1 [file DataSheet_1.docx]
